# A cryopreservation method to recover laboratory- and field-derived bacterial communities from mosquito larval habitats

**DOI:** 10.1371/journal.pntd.0011234

**Published:** 2023-04-05

**Authors:** Serena Y. Zhao, Grant L. Hughes, Kerri L. Coon

**Affiliations:** 1 Department of Bacteriology, University of Wisconsin-Madison, Madison, Wisconsin, United States of America; 2 Departments of Vector Biology and Tropical Disease Biology, Centre for Neglected Topical Disease, Liverpool School of Tropical Medicine, Liverpool, United Kingdom; University of Wisconsin Madison, UNITED STATES

## Abstract

Mosquitoes develop in a wide range of aquatic habitats containing highly diverse and variable bacterial communities that shape both larval and adult traits, including the capacity of adult females of some mosquito species to transmit disease-causing organisms to humans. However, while most mosquito studies control for host genotype and environmental conditions, the impact of microbiota variation on phenotypic outcomes of mosquitoes is often unaccounted for. The inability to conduct reproducible intra- and inter-laboratory studies of mosquito-microbiota interactions has also greatly limited our ability to identify microbial targets for mosquito-borne disease control. Here, we developed an approach to isolate and cryopreserve bacterial communities derived from lab and field-based larval rearing environments of the yellow fever mosquito *Aedes aegypti*–a primary vector of dengue, Zika, and chikungunya viruses. We then validated the use of our approach to generate experimental microcosms colonized by standardized lab- and field-derived bacterial communities. Our results overall reveal minimal effects of cryopreservation on the recovery of both lab- and field-derived bacteria when directly compared with isolation from non-cryopreserved fresh material. Our results also reveal improved reproducibility of bacterial communities in replicate microcosms generated using cryopreserved stocks over fresh material. Communities in replicate microcosms further captured the majority of total bacterial diversity present in both lab- and field-based larval environments, although the relative richness of recovered taxa as compared to non-recovered taxa was substantially lower in microcosms containing field-derived bacteria. Altogether, these results provide a critical next step toward the standardization of mosquito studies to include larval rearing environments colonized by defined microbial communities. They also lay the foundation for long-term studies of mosquito-microbe interactions and the identification and manipulation of taxa with potential to reduce mosquito vectorial capacity.

## Author summary

Mosquitoes develop in the presence of diverse bacterial communities that shape their ability to transmit disease-causing pathogens. However, our current understanding of mosquito-microbiota interactions is largely based on studies of individuals colonized by low-diversity communities of bacteria that are not commonly associated with mosquitoes in the laboratory or field. In this study, we developed an approach to isolate and cryopreserve microbiota from mosquito larval rearing environments in the lab and field. We then demonstrated the successful use of this approach to produce experimental microcosms colonized by standardized microbial communities. Our results are of critical significance to the field of vector biology because they will directly (*i*) facilitate the study of mosquito traits of interest (*e*.*g*., pathogen susceptibility) in the absence of confounding effects of microbiota variation, and (*ii*) enable reproducible intra- and inter-laboratory studies of mosquito-microbiota interactions to identify microbial targets for disease control. Our results are also of broad interest to researchers in the fields of microbial ecology and host-microbe interactions because they demonstrate how tools commonly used to study microbiota assembly and function in mammals can be leveraged in other systems.

## 1. Introduction

The community of bacteria (hereafter referred to as ‘microbiota’) present in the aquatic environments where mosquito larvae develop can have profound impacts on mosquito biology by modulating larval growth and development and, consequently, adult survival, reproduction, and the competency of adult female mosquitoes to transmit human pathogens (reviewed in [1]). However, the enormous diversity and complexity in microbiota composition between different larval rearing environments in both the laboratory and field has to date made it difficult to assign functions to specific community members [1]. The impact of microbiota variation on mosquito phenotypes important for vectorial capacity is also likely driven by interactions with mosquito genotype and environmental factors like diet and temperature [1–3], as is well-documented in vertebrate models [4–7]. However, while experiments are often performed in a controlled and standardized environment using inbred mosquito strains, the microbiota is often not taken into account as a potential source of variation and likely underlies why mosquitoes may respond differently to an intervention in one study compared to another [8–12].

We recently developed an approach to successfully isolate and transfer complete bacterial communities within and between adult mosquitoes of different donor and recipient species, including *Aedes aegypti* and *Culex quinquefasciatus* mosquitoes that serve as primary vectors of arboviral infections and filariasis in humans [13]. We then demonstrated the utility of this approach to study the factors shaping microbiota acquisition and assembly in mosquitoes and the mechanisms by which specific microbial taxa and assemblages contribute to different mosquito traits under controlled conditions [13,14]. However, while these results highlight the value of expanding tools to manipulate the microbiota in mosquitoes, important questions remain regarding (*i*) the utility of microbiota transplantation approaches to establish reproducible communities in mosquito larval rearing environments, which harbor bacterial communities that are much more complex than those in adult mosquitoes [15–19], and (*ii*) how microbiota transplantation efficacy may be shaped by long-term preservation of donor bacterial communities (*e*.*g*., via cryogenic freezing), which is absolutely necessary to facilitate long-term studies and intra- and interlaboratory comparisons but may introduce additional variability via impacts on bacterial viability and recovery [20–25].

In this study, we developed an approach to isolate and cryopreserve microbiota from *Ae*. *aegypti* larval rearing environments in the laboratory and field. We then validated the use of this approach to generate experimental microcosms colonized by standardized lab- and field-derived bacterial communities. Standard centrifugation and freezing methods employing glycerol as a cryoprotectant were used to preserve bacterial cells without any intermediate culturing steps, while high-throughput 16S rRNA gene amplicon sequencing was used to assess recovery and persistence of cryopreserved bacteria in replicate microcosms inoculated at variable cell densities and maintained under conventional mosquito rearing conditions.

## 2. Materials and methods

### (a) Laboratory colony, microbiota isolation and cryopreservation

Microbiota used in this study were derived from a laboratory colony of *Ae*. *aegypti* mosquitoes (Liverpool strain), which is conventionally reared at a constant temperature of 27°C, relative humidity (RH) of 70%, and photoperiod of 16-h light: 8-h dark [26]. In brief, adults are housed in stainless-steel cages (BioQuip, Rancho Dominguez, CA, USA) and provided 10% sucrose in water *ad libitum*. Adult females are blood-fed once per week to promote egg laying using defibrinated sheep blood (Hemostat Laboratories, Dixon, CA, USA) via an artificial membrane feeder. Four days after blood feeding, eggs are collected from colony cages and stored at 27°C and 70% RH. Eggs are then hatched in enamel pans containing deionized water and larvae are fed a standard diet consisting of TetraMin Tropical Flakes Fish Food (Tetra, Melle, Germany) until pupation.

**Fig 1:**
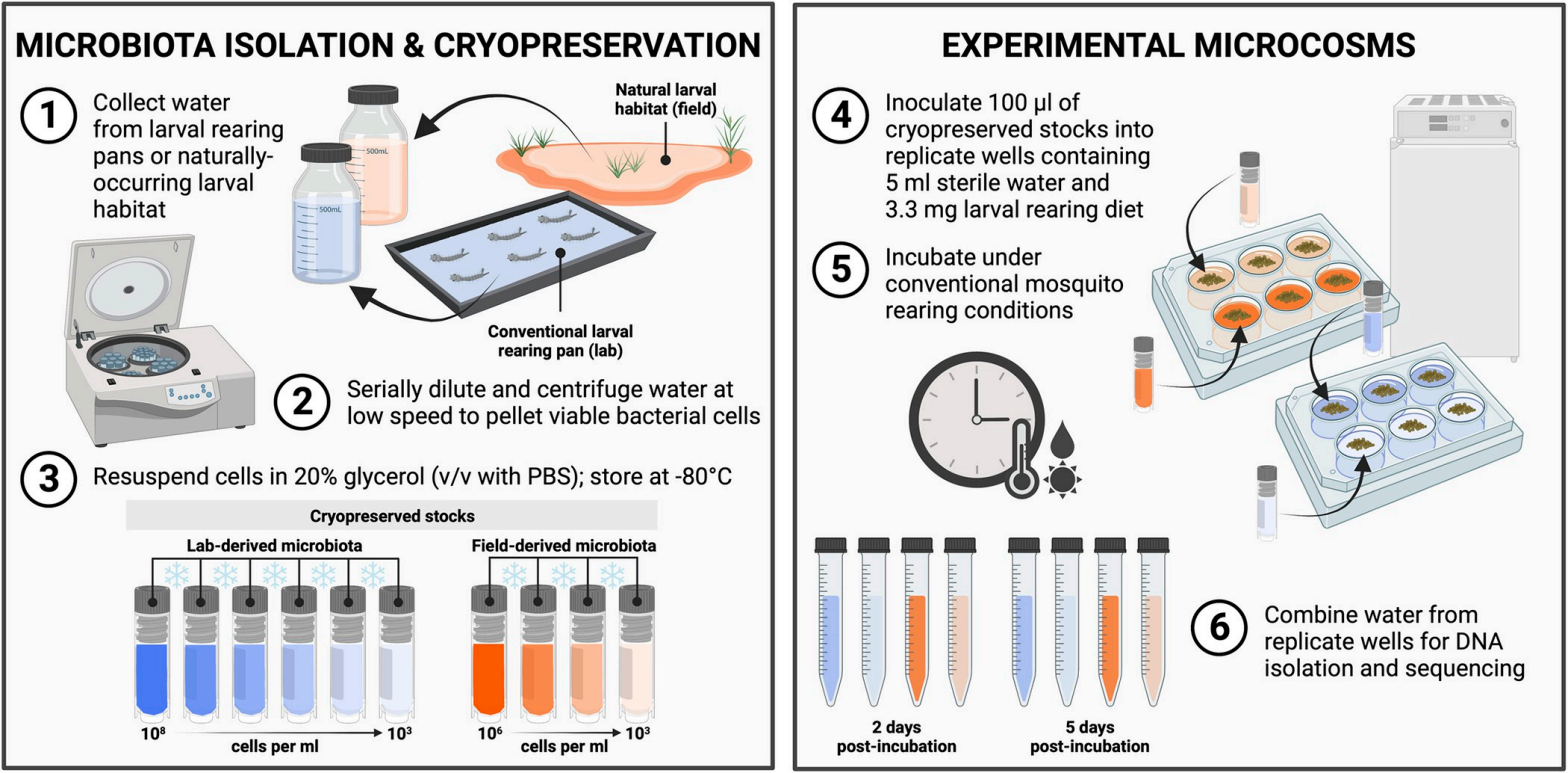
Overview of methodology used to isolate and cryopreserve microbiota and generate experimental microcosms. (*Left*) Isolation and cryopreservation of microbiota from conventional larval rearing pans of our laboratory colony of *Ae*. *aegypti* mosquitoes or a naturally occurring larval habitat in the field. Water from four replicate rearing pans containing larvae that had molted to the final (fourth) instar was collected from the lab, while four aliquots of water from the same larval habitat were collected from the field [1] prior to serial dilution and centrifugation at low speed to pellet any viable bacterial cells [2]. The resulting cell pellets were then resuspended in 20% glycerol (v/v with PBS) and stored at -80°C to produce a total of 40 cryopreserved stocks at variable cell densities (~10^3^−10^8^ bacterial cells per ml) for use in downstream experiments [3]. Aliquots of non-cryopreserved fresh water from each rearing pan or habitat water sample in [1] were also immediately centrifuged at high speed prior to removal of any supernatant and storage of pellets at -20°C for downstream DNA isolation and sequencing to characterize microbiota diversity. (*Right*) Generation of experimental microcosms colonized by standardized bacterial communities. Aliquots of 100 ul from each cryopreserved stock were inoculated into replicate wells of 6-well culture plates containing 5 ml of sterile water and 3.3 mg of a standard larval diet sterilized by gamma irradiation [4]. Replicate wells containing diet and 5 ml of non-cryopreserved fresh water from each rearing pan or habitat water sample in [1] served as unprocessed controls, while wells containing diet and sterile water only served as contamination controls. Culture plates were subsequently incubated under conventional mosquito rearing conditions [5] and water from replicate wells containing the same material was sampled 2- and 5-days post-incubation and combined [6] prior to centrifugation and storage as described above for downstream DNA isolation and sequencing. See “Methods” for more information. Created with BioRender.com.

We isolated the microbiota present in the conventional larval rearing environment of our *Ae*. *aegypti* colony by sampling water from four replicate rearing pans containing larvae that had molted to the final (fourth) instar. For each pan, 500-ml of water was collected in sterile conical tubes (Thermo Fisher Scientific, Waltham, MA, USA) and used to generate the following resources for downstream experiments and sequencing as follows (Fig 1): (*i*) 50-ml of water was immediately centrifuged at high speed (21 130 x *g*) for 20-min, supernatant removed, and pellet stored at -20°C for downstream DNA isolation and sequencing to characterize microbiota diversity, (*ii*) 200-ml of water was temporarily stored (<16-h) unprocessed at 4°C for use in downstream experiments, and (*iii*) the remaining volume (250-ml) was serially diluted, centrifuged at low speed (3220 x *g*) for 20-min, resuspended in 5-ml sterile PBS (1X) containing 20% glycerol, and frozen overnight at -80°C to produce a total of 24 cryopreserved stocks at variable cell densities (~10^8^−10^3^ bacterial cells per ml). Cell density was estimated by counts of colony-forming units (CFU) on R2A agar plates. In brief, aliquots of each cryopreserved microbiota stock were diluted to 10^−4^ and 50-ul of the diluted suspensions were used for plating in triplicate. Cell densities for each stock were then estimated using the average CFU count among replicate plates.

### (b) Field-derived microbiota isolation and cryopreservation

We also isolated the microbiota present in a naturally occurring larval mosquito habitat identified and monitored annually by Public Health Madison & Dane County in Madison, WI USA. In brief, four 500-ml aliquots of water were collected in separate pre-sterilized containers and immediately transported on ice to the laboratory for processing as described above. A total of 16 cryopreserved stocks were produced at variable cell densities (~10^6^−10^3^ bacterial cells per ml). Cell density was estimated as described above.

### (c) Setup and inoculation of experimental microcosms

Sterile 6-well culture plates (Corning, Corning, NY, USA) were used for setting up microcosms (Fig 1). Each microcosm consisted of a single well containing 3.3-mg of a standard mosquito larval rearing diet sterilized by gamma irradiation [3] and: (*i*) 5-ml of unprocessed water derived from either one of four larval rearing pans from our *Ae*. *aegypti* colony or one of four aliquots of water collected from a naturally occurring larval habitat in the field (unprocessed controls), (*ii*) 5-ml of sterile water plus 100-ul of material from a given cryopreserved stock generated from the same water, or (*iii*) 5-ml of deionized water sterilized by autoclaving in the laboratory (contamination control). A total of 300 microcosms were assayed (*n* = 3 wells per water source/treatment). Culture plates were incubated under conventional mosquito rearing conditions (27°C, 70% RH, 16-h light: 8-h dark photoperiod) and 5-ml of water from each well was sampled either 2- or 5-days post-incubation. Water samples collected from replicate wells containing the same water source/treatment were subsequently combined, centrifuged, and stored as described above for downstream DNA isolation and sequencing (Fig 1).

### (d) Bacterial 16S rRNA library construction and sequencing

Total genomic DNA was isolated from a total of 108 samples using a standard phenol-chloroform extraction procedure [27] prior to one-step PCR amplification of the V4 region of the bacterial 16S rRNA gene using barcoded primers as described previously [28]. PCR amplification was performed in 25-ul reactions containing ~10 ng of template DNA, 12.5-ul of 2X HotStart Ready Mix (KAPA Biosystems, Wilmington, MA, USA), and 5-pmol of each primer. No-template reactions as well as reactions using template from blank DNA extractions served as negative controls. Reaction conditions were: initial denaturation at 95°C for 3-min, followed by 25 cycles at 95°C for 30-sec, 58°C for 30-sec, and 72°C for 30-sec, and a final extension step at 72°C for 5-min. Products were visualized on 1% agarose gels and purified using a MagJET NGS Cleanup and Size Selection Kit (Thermo Fisher Scientific, Waltham, MA, USA). The resulting purified libraries were finally quantified using a Quantus fluorometer (Promega) and combined in equimolar amounts prior to paired-end sequencing (2 x 250-bp) on an Illumina MiSeq by the DNA Sequencing Facility at the University of Wisconsin-Madison (Madison, WI, USA). Raw Illumina reads are available in the NCBI Sequence Read Archive (https://www.ncbi.nlm.nih.gov/sra) under BioProject ID PRJNA856768.

### (e) Sequence analysis

De-multiplexed reads were processed using the DADA2 pipeline in QIIME 2–2021.2 [29,30] to infer exact amplicon sequence variants (ASVs). In brief, sequence reads were first filtered using DADA2’s recommended parameters. Filtered reads were then de-replicated and denoised using default parameters. After building the ASV table and removing chimeras, taxonomy was assigned using a Naïve Bayes classifier natively implemented in QIIME and pre-trained against the Silva reference database (138) [31]. A phylogenetic tree was built using FastTree 2 [32] from a multiple sequence alignment made with the MAFFT alignment tool [33] against the Silva core reference alignment [31]. All endpoint artifacts generated in QIIME were then exported, merged with metadata, and converted to a phyloseq object for further analysis in R (http://www.r-project.org/) [34]. Rooting of the phylogenetic tree was performed in R using phyloseq and a decontamination procedure was implemented using a two-tiered approach implemented in the R package ‘decontam’ [35]. In brief, DNA quantification values prior to library pooling in study samples, blank DNA extraction products, and PCR negative controls were used to generate a list of likely contaminant reads. Contaminant reads that were more prevalent in control samples than in study samples were then removed from the entire dataset, along with samples with fewer than 100 total reads and reads classified as ‘Archaea’, ‘Chloroplast’, or ‘mitochondria’ prior to downstream analyses.

Patterns of alpha diversity (as measured by Shannon’s H index and ASV richness) and beta diversity (as measured by the Bray-Curtis dissimilarity index) were analyzed using the R package ‘vegan’ [36]. Differences in alpha and beta diversity between water samples collected from either conventional larval rearing pans or the larval habitat in the field versus experimental microcosms were analyzed by Bonferroni-corrected pairwise Dunn’s tests to compare microcosm samples to their respective microbiota source (*i*.*e*., larval rearing pan or habitat water sample). The average relative abundance of bacterial taxa present in rearing pan or habitat water samples that were detected in associated microcosm samples was then determined as follows: (*i*) if an ASV was present in a given rearing pan or habitat water sample and at least one of its associated microcosm samples, that ASV was assumed to be shared; ASVs present in a given rearing pan or habitat water sample that were absent across all associated microcosm samples were considered unique to that rearing pan or habitat water sample; (*ii*) reads corresponding to shared ASVs were then summed for each rearing pan and habitat water sample and divided by the total number of reads (*i*.*e*., shared plus unique) generated from the same sample; these calculations produced a total of four shared relative abundance estimates for each microbiota source.

Differences in the proportion of rare and common taxa present in different microcosm samples were analyzed by Bonferroni-corrected Fisher’s exact tests. The significance of sample clustering by water source/treatment and time of sampling was analyzed by permutational multivariate analysis of variance (PERMANOVA), followed by *post-hoc* pairwise permutation tests for homogeneity of multivariate dispersions (PERMDISP). The differential abundance of ASVs between larval rearing pans and different experimental microcosm sample groups was analyzed based on a Wilcoxon rank sum test and Welch’s t test using the R package ALDEx2 [37–39]. In order to identify ASVs significantly and systematically responding to cryopreservation, the ‘denom’ argument was set to “zero” to account for the complete loss of some taxa in samples derived from microcosms containing cryopreserved microbiota while allowing for all taxa present in at least one rearing pan or habitat water sample to be included in the analysis. To determine the effect of cryopreservation on bacterial populations, the effect size was calculated, which is the median of the ratio of the between group difference and the larger of the variances within groups. The Benjamini-Hochberg-Yekutieli procedure was then used to account for multiple testing, and corrected values were expressed as false discovery rates (FDR) [40]. Finally, statistically significant differences in the average relative abundance of reads from shared taxa among replicates of different sample groups were assessed using Bonferroni-corrected pairwise Dunn’s tests followed by pairwise Fligner-Killeen tests to assess the homogeneity of variances. The same tests were then used to compare patterns of alpha and beta diversity among replicates of the same sample groups. Input files for the QIIME pipeline as well as raw data files and R code for statistical analyses have been deposited in the Dryad Digital Repository [https://doi.org/10.5061/dryad.dfn2z354z] [41].

## 3. Results

### (a) Impact of cryopreservation on recovery of bacteria from laboratory-derived larval rearing pans

We initially sought to develop an approach to isolate and cryopreserve microbiota from conventional larval rearing pans in the laboratory for recapitulation in experimental microcosms. Multiplex sequencing of 16S rRNA gene amplicons for the resulting rearing pan- and microcosm-derived water samples generated a total of 2,669,979 quality-filtered reads with a median sequencing depth of 44,707 reads per sample (S1 Table). An unusually low number of reads (<100) were obtained for two samples, which were removed from subsequent analyses (S1 Table). Rarefaction curves for the remaining samples saturated at ~500 sequences, indicating that most (if not all) bacteria in each sample were captured (S1 Fig).

**Fig 2:**
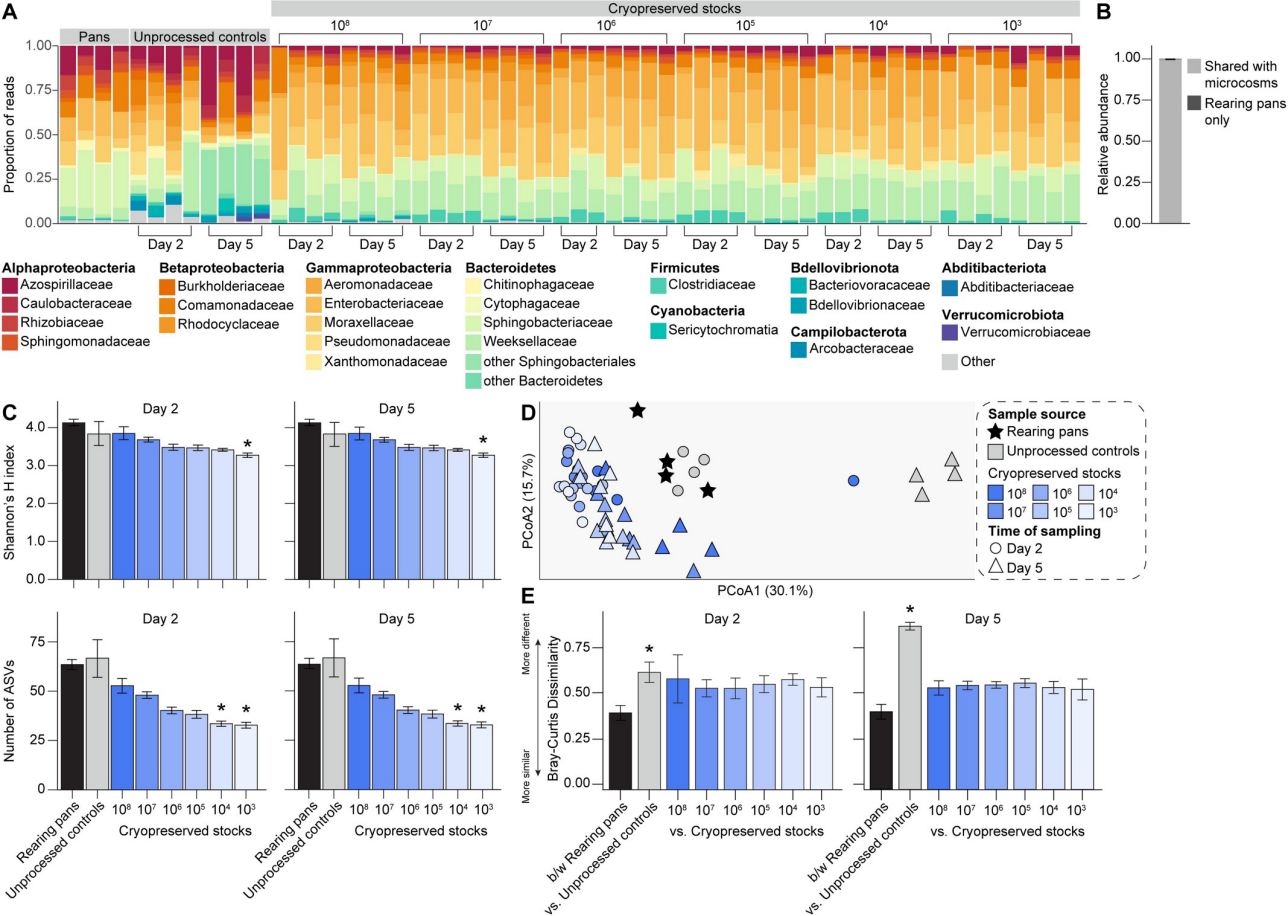
Bacterial diversity in laboratory larval rearing pans and experimental microcosms generated using lab-derived microbiota. (A) Relative abundance of bacterial families in water sampled from: (*i*) conventional rearing pans containing fourth instar larvae from our standard *Ae*. *aegypti* laboratory colony, (*ii*) experimental microcosms containing unprocessed water from the same rearing pans, or (*iii*) experimental microcosms containing sterile water plus material from a given cryopreserved stock. Each bar presents the proportion of sequencing reads assigned to a given bacterial family. Low abundance families (<1%) are represented by the ‘Other’ category. (B) Average relative abundance of rearing pan microbiota shared with experimental microcosms (mean value ± standard error is shown). (C) Alpha diversity of rearing pans and experimental microcosms, as measured by Shannon’s H index (top) and ASV richness (bottom). Mean values ± standard errors are shown. Asterisks (*) indicate significant differences between a given group of experimental microcosm samples relative to rearing pan samples (Dunn’s test with Bonferroni correction, *P* < 0.01). (D) Principal coordinates analysis using the Bray-Curtis dissimilarity index. Symbols are colored by sample source (rearing pans, black; experimental microcosms containing unprocessed water, grey; experimental microcosms containing sterile water plus material from a given cryopreserved stock, blue). Time of sampling (Day 2 or Day 5) of experimental microcosms is designated by symbol shape. (E) Average Bray-Curtis dissimilarity between (b/w) rearing pans versus between a given rearing pan and group of experimental microcosm samples. Mean values ± standard errors are shown. Asterisks (*) indicate comparisons for which the average dissimilarity between a given rearing pan and group of experimental microcosm samples was significantly higher than that expected as a result of the microbiota isolation and cryopreservation procedure itself (*i*.*e*., between rearing pans) (Dunn’s test with Bonferroni correction, *P* < 0.05).

We identified 94 ASVs across the conventional larval rearing pans we sampled. However, the vast majority (>92%) of reads from these samples were assigned to ASVs belonging to one of eight bacterial families within the following phyla/classes: Alphaproteobacteria (Azospirillaceae, Rhizobiaceae, Sphingomonadaceae), Betaproteobacteria (Comamonadaceae), Gammaproteobacteria (Enterobacteriaceae, Moraxellaceae), and Bacteroidetes (Sphingobacteriaceae, Weeksellaceae) (Fig 2A). A total of 85 of the 94 ASVs found in rearing pans, representing >99% of all rearing pan sequences, were also detected in the experimental microcosms we set-up and sampled (Fig 2B), although recovery varied with respect to the time of microcosm sampling, the cell density of the cryopreserved stock used to generate a given microcosm, and how common a given ASV was across the rearing pans we sampled and sequenced (S2 Fig). Cryopreservation had the greatest impact on recovery of rare ASVs (*i*.*e*., those with a maximum relative abundance ≤1% in rearing pans), with significantly fewer rare ASVs being recovered in experimental microcosms generated using cryopreserved stocks of lower cell densities (10^4^, 10^5^), even at 5 days post-incubation (S2 Fig). However, there were no significant differences in recovery of more common ASVs (*i*.*e*., those with a minimum relative abundance >1% in rearing pans) between any of the microcosms we generated (S2 Fig).

### (b) Recapitulation of bacterial diversity in experimental microcosms

Experimental microcosms generated using material from cryopreserved stocks also generally contained bacterial communities that did not significantly differ in alpha diversity from the communities present in the rearing pans we initially sampled, with the only exceptions again being those generated using cryopreserved stocks of lower cell densities (10^4^, 10^5^) (Fig 2C and S1 Table). Further, while a principal coordinates analysis using the Bray-Curtis dissimilarity index identified significant clustering by sample source (*i*.*e*., rearing pans vs. experimental microcosms containing unprocessed water or sterile water plus material from cryopreserved stocks; PERMANOVA, *P* = 0.001) (Fig 2D), *post-hoc* pairwise permutation tests for homogeneity of multivariate dispersions revealed significantly higher dispersion values amongst experimental microcosms containing unprocessed water than amongst microcosms containing sterile water plus material from cryopreserved stocks or the larval rearing pans (PERMDISP, *P* = 0.001). Differences in beta diversity, measured as average Bray-Curtis dissimilarity, were also overall higher between rearing pans and unprocessed controls than between rearing pans and experimental microcosms generated using material from cryopreserved stocks, regardless of cell density (Fig 2E).

### (c) Changes in the relative abundance of specific taxa in response to cryopreservation

**Fig 3:**
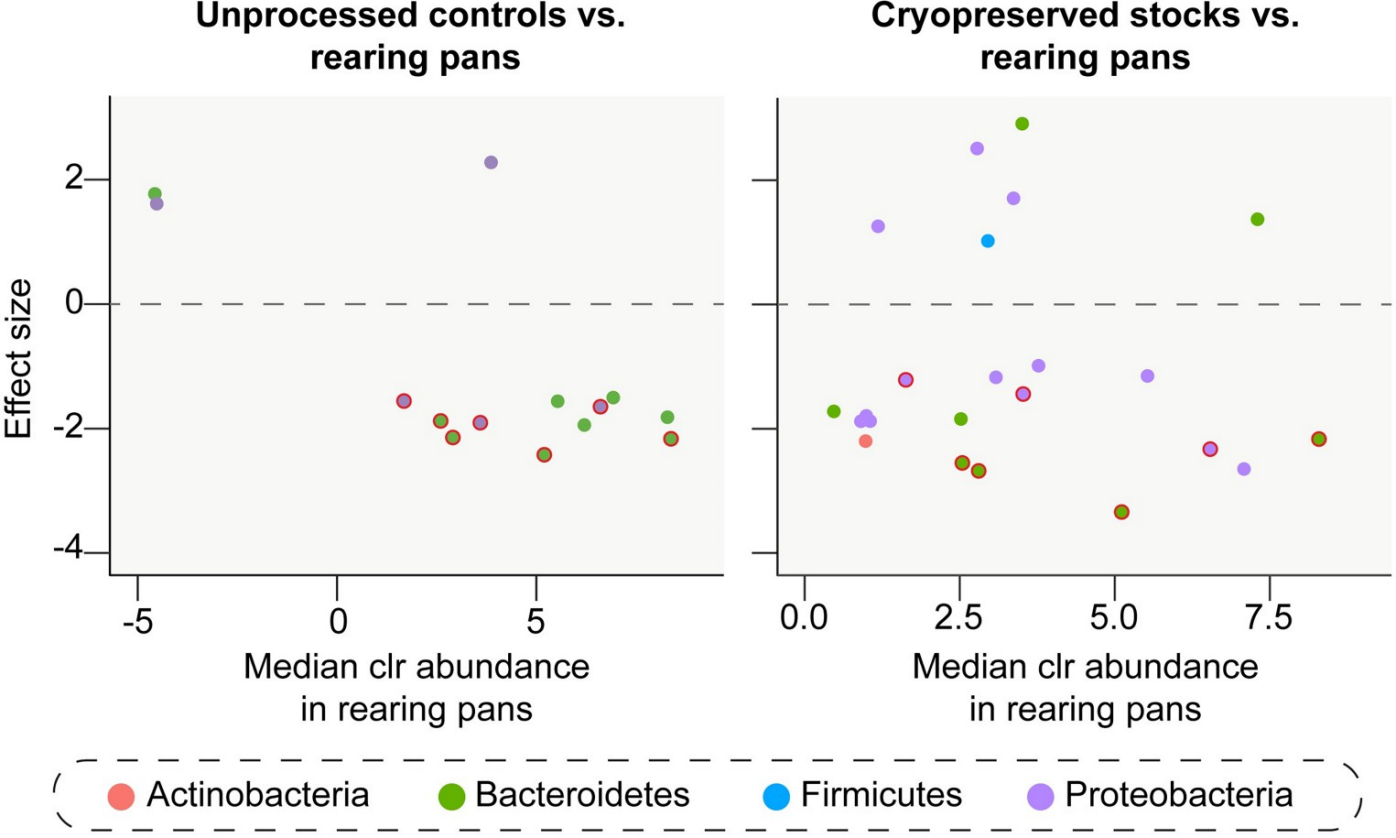
ASVs that significantly varied in abundance between larval rearing pans and experimental microcosms containing unprocessed water (*left*) or sterile water plus material from cryopreserved stocks (*right*) (ALDEx2, *P* < 0.05; FDR, *P* < 0.05). Plots show the median centered log-ratio (clr) value for each ASV across larval rearing pans (*x* axis) and the effect of re-culturing with or without cryopreservation under conventional mosquito rearing conditions (*y* axis). An effect size < 0 indicates that the ASV abundance significantly decreased between rearing pans and a given group of experimental microcosm samples, and an effect size of > 0 indicates that the ASV abundance significantly increased. ASVs are colored by phylum (see legend). ASVs with a bold red outline were differentially abundant in both experimental microcosms containing unprocessed water and experimental microcosms containing sterile water plus material from cryopreserved stocks.

Finally, we performed ALDEx2 tests to identify rearing pan ASVs that significantly changed in relative abundance in response to cryopreservation and/or re-culturing under conventional mosquito rearing conditions (Figs 3 and S3 and S4). These tests revealed that 30 of the 94 ASVs found in rearing pans, classified as members of one of four bacterial phyla (Actinobacteria, Bacteroidetes, Firmicutes, and Proteobacteria), showed significantly different relative abundances in experimental microcosms containing unprocessed water and/or sterile water plus material from cryopreserved stocks (Figs 3 and S3 and S4). However, only 10 of these ASVs, representing <7% of all rearing pan sequences, were specifically negative affected (*i*.*e*., decreased in abundance) in response to cryopreservation (Figs 3 and S3 and S4). The vast majority (>94%) of rearing pan ASVs recovered in microcosms containing material from cryopreserved stocks also persisted over time, with only four ASVs showing significantly lower relative abundances in samples collected on Day 5 when compared to those collected on Day 2 post-incubation (ALDEx2, *P* < 0.05; FDR, *P* < 0.05).

### (d) Validation of isolation and cryopreservation approaches using field-derived microbiota

Next we sought to validate the use of cryopreservation approaches developed above to generate experimental microcosms colonized by microbiota derived from a naturally occurring mosquito larval habitat in the field. Multiplex sequencing of 16S rRNA gene amplicons for the resulting habitat- and microcosm-derived water samples generated a total of 2,111,045 quality-filtered reads with a median sequencing depth of 43,541 reads per sample (S2 Table), with rarefaction curves for most samples saturating at ~3,000 sequences (S5 Fig).

**Fig 4:**
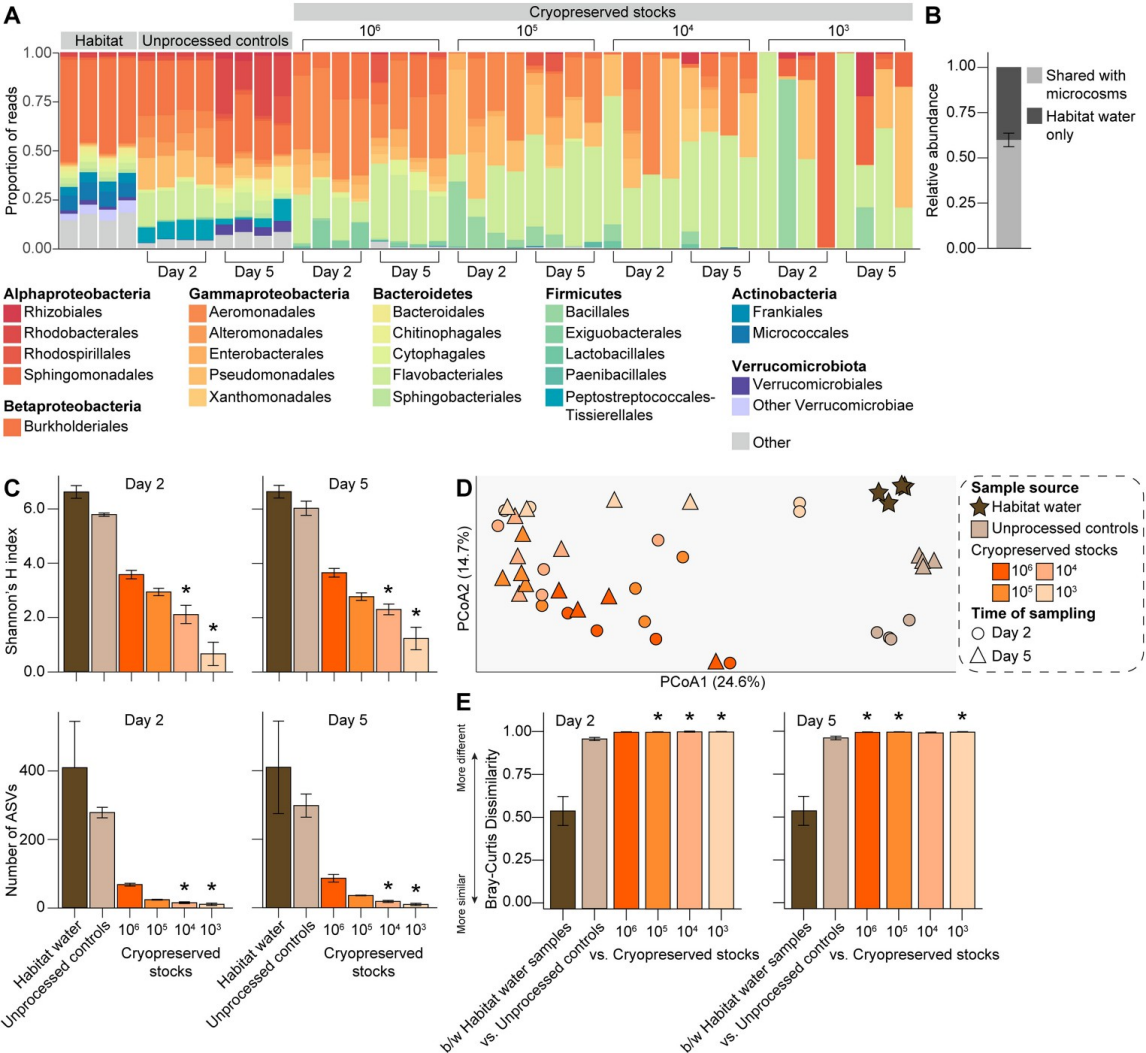
Bacterial diversity in a natural larval habitat and experimental microcosms generated using field-derived microbiota. (A) Relative abundance of bacterial orders in water sampled from: (*i*) a naturally occurring larval mosquito habitat in the field, (*ii*) experimental microcosms containing unprocessed water from the same habitat, or (*iii*) experimental microcosms containing sterile water plus material from a given cryopreserved stock. Each bar presents the proportion of sequencing reads assigned to a given bacterial family. Low abundance orders (<1%) are represented by the ‘Other’ category. (B) Average relative abundance of habitat water microbiota shared with experimental microcosms (mean value ± standard error is shown). (C) Alpha diversity of habitat water samples and experimental microcosms, as measured by Shannon’s H index (top) and ASV richness (bottom). Mean values ± standard errors are shown. Asterisks (*) indicate significant differences between a given group of experimental microcosm samples relative to habitat water samples (Dunn’s test with Bonferroni correction, *P* < 0.05). (D) Principal coordinates analysis using the Bray-Curtis dissimilarity index. Symbols are colored by sample source (habitat water, brown; experimental microcosms containing unprocessed water, tan; experimental microcosms containing sterile water plus material from a given cryopreserved stock, orange). Time of sampling (Day 2 or Day 5) of experimental microcosms is designated by symbol shape. (E) Average Bray-Curtis dissimilarity between (b/w) habitat water samples versus between a given habitat water sample and group of experimental microcosm samples. Mean values ± standard errors are shown. Asterisks (*) indicate comparisons for which the average dissimilarity between a given habitat water sample and group of experimental microcosm samples was significantly higher than that expected as a result of the microbiota isolation and cryopreservation procedure itself (*i*.*e*., between habitat water samples) (Dunn’s test with Bonferroni correction, *P* < 0.05).

As expected, we identified a substantially higher diversity of bacteria (993 ASVs) across the field-derived water samples as compared to those derived from conventional larval rearing pans in the laboratory, with ~42% of reads being assigned to ASVs belonging to the eight bacterial families dominating communities in rearing pans and the remaining ~58% being assigned to ASVs belonging to one of 306 other families within 187 bacterial orders and 34 phyla/classes (Fig 4A). A substantially higher diversity of bacteria (182 ASVs) was also detected in the experimental microcosms we set-up and sampled, although overall recovery was significantly lower with respect to the relative abundance of habitat ASVs that were shared with experimental microcosms (Fig 4B) and impacts on alpha diversity were more marked in microcosms generated using cryopreserved stocks of higher cell densities (Fig 4C and S2 Table) than previously observed for cryopreserved stocks of lab-derived microbiota (Fig 2C and S1 Table). Differences in beta diversity, measured as Bray-Curtis dissimilarity, were also overall higher between habitat samples and microcosms containing material from cryopreserved stocks (Fig 4D and 4E) than previously observed for lab-derived bacterial communities (Fig 2D and 2E), although no dispersion effects were detected (PERMDISP, *P* = 0.426).

**Fig 5:**
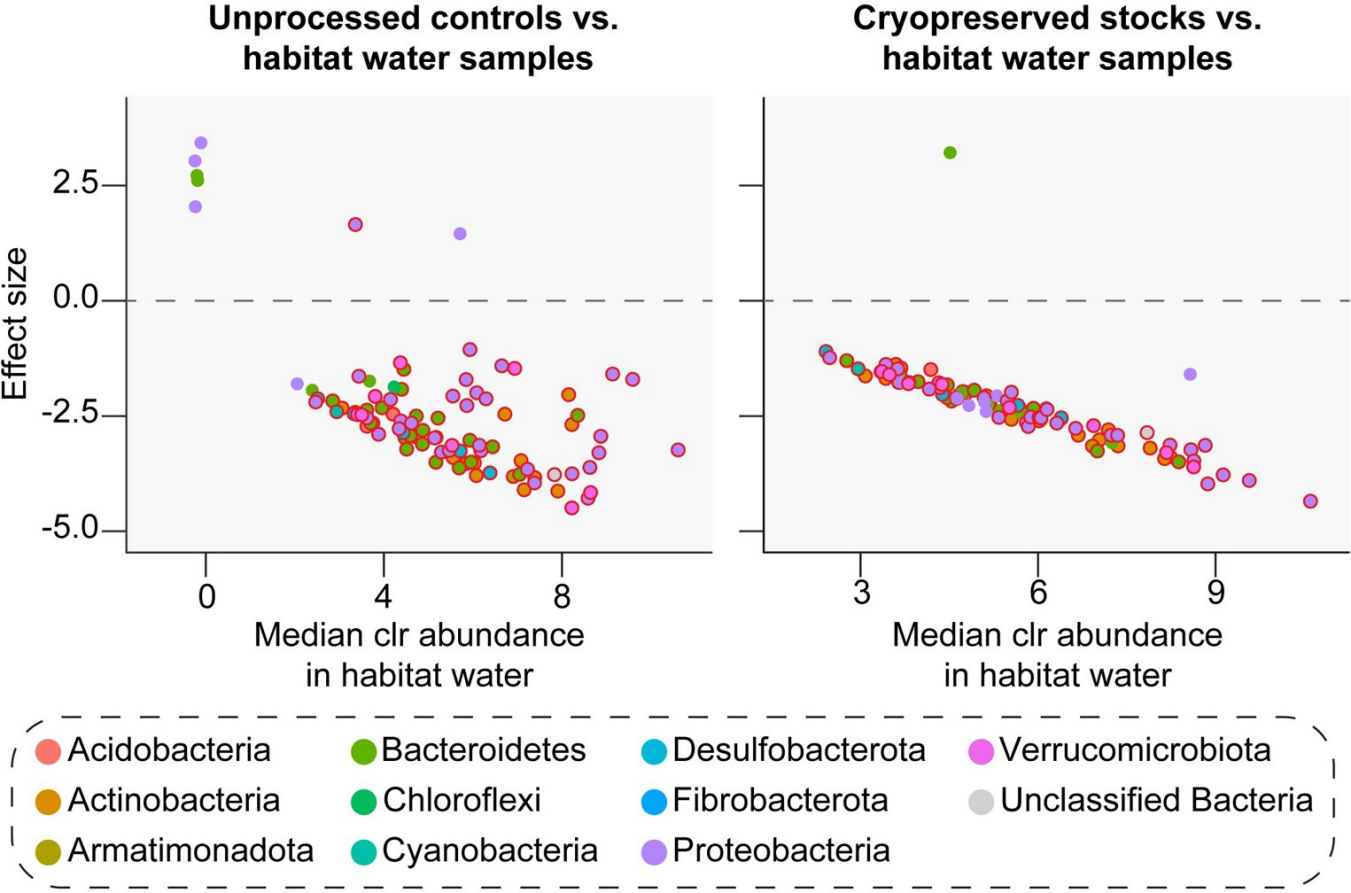
ASVs that significantly varied in abundance between habitat water samples and experimental microcosms containing unprocessed water (*left*) or sterile water plus material from cryopreserved stocks (*right*) (ALDEx2, *P* < 0.05; FDR, *P* < 0.05). Plots show the median centered log-ratio (clr) value for each ASV across habitat water samples (*x* axis) and the effect of re-culturing with or without cryopreservation under conventional mosquito rearing conditions (*y* axis). An effect size < 0 indicates that the ASV abundance significantly decreased between habitat water samples and a given group of experimental microcosm samples, and an effect size of > 0 indicates that the ASV abundance increased. ASVs are colored by phylum (see legend). ASVs with a bold red outline were differentially abundant in both experimental microcosms containing unprocessed water and experimental microcosms containing sterile water plus material from cryopreserved stocks.

ALDEx2 tests identified a total of 110 of the 993 ASVs found in the habitat water samples we sequenced showing significantly different relative abundances in experimental microcosms, but only 12 of these ASVs, representing <6% of all habitat water sequences, were specifically negatively affected in response to cryopreservation (Figs 5 and S6 and S7). In contrast, 87 ASVs, representing ~68% of all habitat water sequences, were negatively affected in both experimental microcosms containing unprocessed water and microcosms containing material from cryopreserved stocks (Figs 5 and S6 and S7). However, similar to our ALDEx2 results for microcosms containing lab-derived microbiota, all (100%) of the habitat ASVs recovered in microcosms generated using cryopreserved stocks containing field-derived microbiota persisted over time, with no ASVs showing significantly lower relative abundances between samples collected on Day 5 and Day 2 post-incubation (ALDEx2, *P* > 0.05).

### (e) Reproducibility of bacterial communities in experimental microcosms

**Fig 6:**
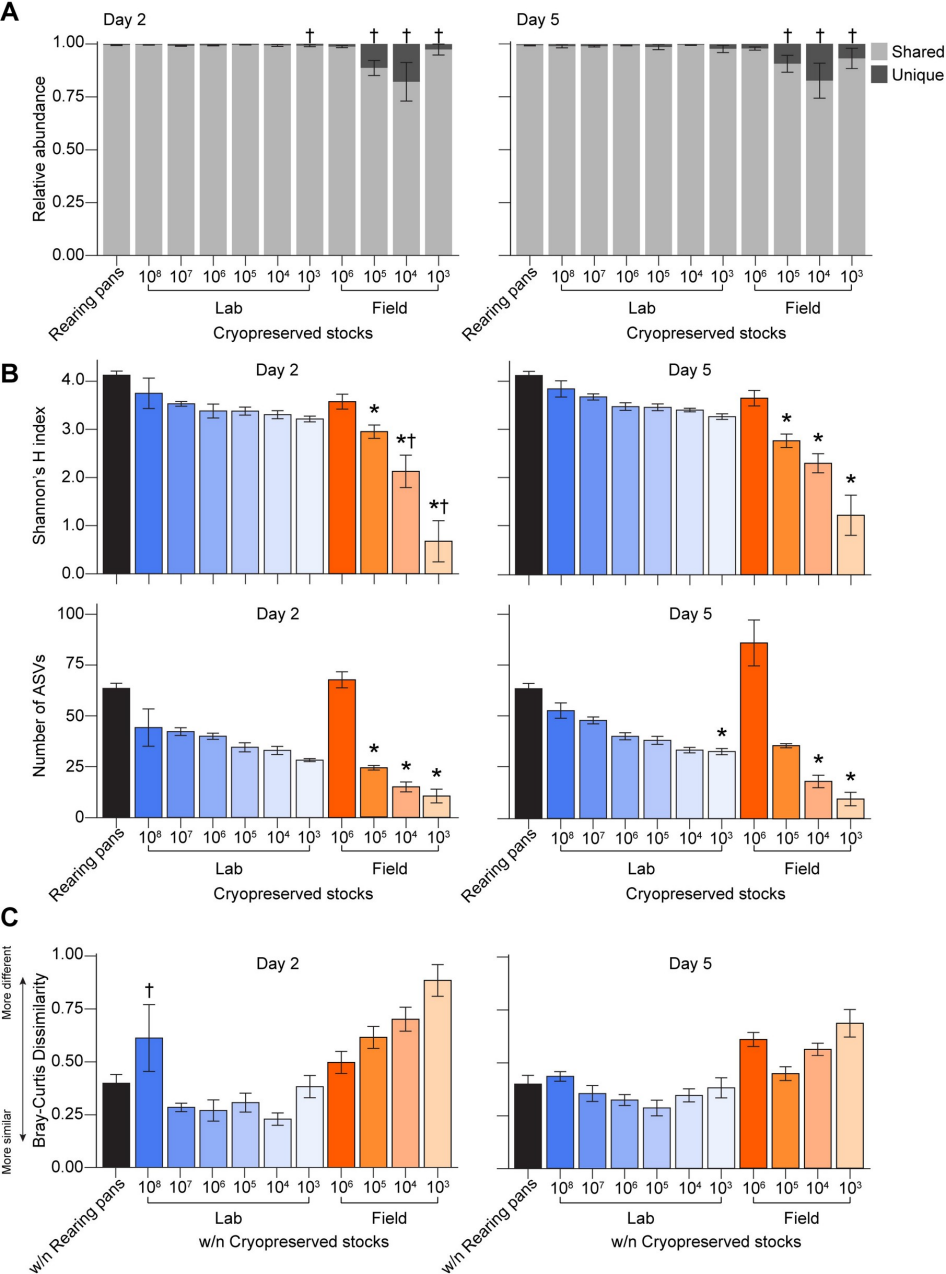
Reproducibility of bacterial communities in replicate microcosms. (A) Average relative abundance of shared microbiota among rearing pan samples and replicates of a given group of experimental microcosms. Daggers (†) indicate comparisons for which the variance of a given group of experimental microcosm samples was significantly higher than that of rearing pan samples (Fligner-Killeen test, *P* < 0.05). No significant differences in averages were detected between rearing pan samples and any experimental microcosm sample group (Dunn’s test with Bonferroni correction, *P* > 0.05). (B) Alpha diversity of rearing pans and experimental microcosms generated using cryopreserved stocks of lab- and field-derived microbiota, as measured by Shannon’s H index (top) and ASV richness (bottom). Mean values ± standard errors are shown. Asterisks (*) indicate significant differences between a given group of microcosm samples relative to rearing pan samples (Dunn’s test with Bonferroni correction, *P* < 0.05). Daggers (†) indicate comparisons for which the variance of a given group of experimental microcosm samples was significantly higher than that of rearing pan samples (Fligner-Killeen test, *P* < 0.05). (C) Average Bray-Curtis dissimilarity within (w/n) rearing pans versus within a given group of experimental microcosm samples. Mean values ± standard errors are shown. Daggers (†) indicate comparisons for which the variance of a given group of experimental microcosm samples was significantly higher than that of rearing pan samples (Fligner-Killeen test, *P* < 0.01). No significant differences in averages were detected between rearing pan samples and any experimental microcosm sample group (Dunn’s test with Bonferroni correction, *P* > 0.05).

While our isolation and cryopreservation approaches resulted in the loss of some lab- and field-derived bacterial taxa and concurrent changes in the total alpha and beta diversity of microbiota within experimental microcosms as compared to the larval rearing pans and habitat water we originally sampled, we observed little variation among the bacterial communities present within replicate microcosms generated using the same cryopreserved stock, with the only exceptions being those generated using stocks of lower cell densities (Fig 6). On average, replicate microcosms shared 30 taxa, which accounted for ~96% of their total reads–consistent with the amount of bacteria shared among larval rearing pans in the laboratory (Fig 6A). Bacteria introduced into replicate microcosms also assembled into communities that overall exhibited similar patterns of inter-replicate alpha and beta diversity as those observed in rearing pans under conventional rearing conditions (Fig 6B and 6C).

## 4. Discussion

Mosquitoes live in close association with bacteria and other microorganisms that shape their ability to transmit pathogens [1]. However, the immense diversity and variability of the microbiota within and between different populations of mosquitoes in the laboratory and field have made studying mosquito-microbe interactions–and identifying bacteria that reduce the vectorial capacity of mosquitoes–a formidable challenge [1]. As in other animals, numerous factors have the potential to shape variation in mosquito microbiota and therefore variation in mosquito phenotypes, including the microbiota present in the aquatic environment in which larvae develop, environmental conditions (e.g., diet, temperature), and host genetics [2,3,10,11,15–19,42–50]. However, while experiments with mosquitoes are commonly conducted using genetically identical individuals under highly controlled environmental conditions, tools to standardize the microbiota present in the larval rearing environment are comparatively limited [3,13,51–54].

In this study, we first developed an approach to isolate and cryopreserve microbiota from conventional larval rearing pans in the laboratory for recapitulation in experimental microcosms. We intentionally conducted our experiments in the absence of any larvae in order to: (*i*) decouple the effects of sample processing and the cell density of cryopreserved stocks on the recovery and persistence of bacteria in microcosms from those of larvae, which are known to shape water pH, nutrient levels, and even bacterial metabolism [55,56], and (*ii*) better reflect natural microbiota exposure and acquisition processes in the field, whereby larvae hatch from eggs laid directly into (or adjacent to) water containing established bacterial communities [57–59]. High-throughput sequencing of 16S rRNA gene amplicons from water collected from four replicate rearing pans from our laboratory colony of *Ae*. *aegypti* revealed a bacterial community comprised of ~100 unique bacterial taxa and dominated by members of the Proteobacteria, Bacteroidetes, and Firmicutes, consistent with sequencing studies of other laboratory colonies of mosquitoes [1,3,15,17,50,60]. The vast majority of this bacterial diversity was also recovered in both experimental microcosms generated using non-cryopreserved fresh material and experimental microcosms generated using cryopreserved material from the same rearing pans, regardless of stock cell density. The only exceptions were a handful of representatives of the class Alphaproteobacteria, members of the orders Burkholderiales, Chitinophagales, and Sphingobacteriales, as well as one very rare ASV belonging to the genus *Paenarthrobacter*, which were significantly reduced or absent in experimental microcosms and may represent taxa that are not readily amenable to centrifugation and/or cryopreservation using our methodology.

We next validated our isolation and cryopreservation approach using microbiota derived from water collected from a naturally occurring larval habitat in the field. As expected, the bacterial community present in this habitat was comprised of a substantially higher diversity of ~1,000 unique taxa, although these taxa were dominated by members of the same phyla (Proteobacteria, Bacteroidetes, Firmicutes, Actinobacteria) detected in laboratory rearing pans and commonly detected in field-collected mosquitoes [2,10,11,15–19,42–49,60–65]. Recovery of field-derived taxa in experimental microcosms generated using cryopreserved material from the same habitat was also overall much lower than what we observed in microcosms generated using cryopreserved stocks of lab-derived microbiota, although the taxa lost or significantly reduced in microcosms generated using cryopreserved material were consistent with those lost or significantly reduced in microcosms generated using fresh material–strongly suggesting that the observed shifts in alpha and beta diversity in microcosms were not the result of cryopreservation but rather the inability of many field-derived taxa to thrive under conventional mosquito rearing conditions in the laboratory. This, combined with our results using lab-derived microbiota, strongly suggests that our isolation and cryopreservation procedure was sufficient to conserve most bacteria. Nevertheless, future studies could compare the results here to results obtained using different long-term preservation approaches, including periodic subculturing, drying, freeze-drying, and cryopreservation with different cryoprotectants at variable concentrations [20,22,23,66–71]. Future work will also be necessary to confirm that the patterns observed here are the same for experimental microcosms generated using material from cryopreserved stocks stored for longer periods of time [72] and to formally assess the impact of long-term cryopreservation and resuscitation on cell viability and functional stability [73,74]. Additionally, it would be important to determine that the cryopreservation approach is appropriate for microbiota collected from diverse environments given the variation between different habitats [2,15,16,19,42–44,47–49].

The cell density of cryopreserved stocks also had a much larger impact on the recovery and persistence of field-derived taxa, with microcosms generated using stocks of lower cell densities exhibiting more variable bacterial communities that were overall lower in complexity that communities in microcosms generated using stocks of higher cell densities. This is consistent with results showing that small sampling volumes from aquatic and other high-complexity environments like soil often fail to adequately capture bacterial diversity [75–77], owing to microscale heterogeneity and bottleneck effects during population sub-sampling that leads to the stochastic proliferation of some taxa and the loss of others [78–81].

Overall, experimental microcosms generated using cryopreserved stocks containing lab-derived microbiota exhibited levels of reproducibility that were the same or better than those present under conventional rearing conditions in the laboratory, highlighting the value of our approach for intra-and inter-lab studies of mosquitoes in the presence of standardized microbial communities. Similar levels of reproducibility could also be replicated in microcosms generated using cryopreserved stocks of field-derived microbiota, although reproducibility significantly decreased with decreasing inoculum size–consistent with our previous observation that variability among the bacterial communities present within replicate microcosms was significantly higher in those generated using cryopreserved stocks of lower cell densities. Future studies are warranted to establish best practices for field habitat sampling, though our results at minimum suggest that higher-volume samples are preferable, and that sampling should be avoided in situations where bacterial density is expected to be low (*e*.*g*., immediately after a rainfall). Defined diets that support mosquito growth and development could also be developed to improve the reproducibility of field bacterial diversity between replicate microcosms, including the maintenance of rare taxa important for community assembly and stability [82,83].

Owing to their ability to impact numerous components of mosquito vectorial capacity, there is a growing interest in exploiting microbes for mosquito-borne disease control. For example, bacteria that naturally colonize the mosquito gut could be genetically modified to produce effector molecules that alter the mosquito’s ability to become infected with and transmit pathogens, or that reduce mosquito fecundity or lifespan (*i*.*e*., paratransgenesis). Unmodified bacteria that naturally inhibit pathogen colonization or mosquito fitness could also be disseminated to mosquito populations. The identification of suitable microbial candidates for pathogen or mosquito control will require a comprehensive understanding of the factors that influence the acquisition, maintenance, and transmission of mosquito microbiota and the mechanisms that underlie how individual microbial species and assemblages impact mosquito vectorial capacity. However, the dearth of tools to manipulate mosquito microbiota, including microbiota derived from naturally occurring mosquito habitats in the field, has greatly slowed progress in this area. In this way, the results reported here not only provide a critical first step toward the standardization of microbial inputs in mosquito studies, but also provide a critical first step toward the identification of taxonomic and functional profiles of bacteria associated with phenotypic traits of interest in mosquitoes. For example, a defined microbiota could be universally adopted by the community to conduct vector competence assays in the absence of confounding effects due to microbiota variation. Libraries of cryopreserved microbiota could also be screened to identify bacteria that improve or reduce mosquito fitness and/or pathogen susceptibility, or to predict the success of individual microbial candidates under variable microbial conditions. Similarly, the results reported here strongly support that our methods could immediately be leveraged to expand studies of mosquito-microbiota interactions to include microbial genotypes derived from the field, and thereby conduct lab-based mosquito experiments with field-relevant microbiota.

## Supporting information

S1 Table Sequencing and diversity statistics for 16S rRNA gene amplicon libraries prepared from water collected from conventional larval rearing pans in the laboratory and resulting experimental microcosms. Asterisks (*) indicate samples that were removed from the dataset prior to downstream analyses.
(DOCX)

S2 Table Sequencing and diversity statistics for 16S rRNA gene amplicon libraries prepared from water collected from a naturally occurring mosquito larval habitat in the field and resulting experimental microcosms. (DOCX)

S1 Fig Rarefaction data from Illumina sequencing of water from conventional larval rearing pans in the laboratory and resulting experimental microcosms. Reads from each water library were sampled starting at 1 sequence per step and increased in increments of 100 until the total number of reads per sample was reached. Lines are colored by sample source (rearing pans, black; experimental microcosms containing unprocessed water, grey; experimental microcosms containing sterile water plus material from a given cryopreserved stock, blue). Time of sampling of experimental microcosms is designated by line type (Day 2, long-dash; Day 5, dot-dash).
(TIF)

S2 Fig Proportion of rare (left) and abundant (right) ASVs found in at least one larval rearing pan that were detected in experimental microcosms containing unprocessed water or sterile water plus material from a given cryopreserved stock. An ASV was considered “rare” if it had a maximum relative abundance ≤1% across the four larval rearing pans we sampled, while ASVs with a minimum relative abundance >1% were considered “abundant”. Asterisks (*) indicate significant differences between experimental microcosms generated using cryopreserved stocks relative to unprocessed controls as determined by paired Fisher’s exact tests with Bonferroni correction (*P* < 0.05).
(TIF)

S3 Fig Differentially abundant ASVs in water collected from conventional larval rearing pans in the laboratory and experimental microcosms containing unprocessed pan water, identified through ALDEx2 testing (ALDEx2, *P* < 0.05; FDR, *P* < 0.05). Each ASV is presented with its lowest annotated taxonomic rank (to genus level) together with its ASV ID. The ASVs are color-coded according to the phyla they belong to and plotted according to their effect size, calculated as the levels in samples from experimental microcosms relative to levels in samples from larval rearing pans. Dot sizes correspond to the median centered log-ratio (clr) abundance value for each ASV across rearing pan samples. Dots with a bold red outline represent ASVs that were differentially abundant in both experimental microcosms containing unprocessed water and experimental microcosms containing sterile water plus material from cryopreserved stocks (see S4 Fig).
(TIF)

S4 Fig Differentially abundant ASVs in water collected from conventional larval rearing pans in the laboratory and experimental microcosms containing sterile water plus material from cryopreserved stocks, identified through ALDEx2 testing (ALDEx2, *P* < 0.05; FDR, *P* < 0.05). Each ASV is presented with its lowest annotated taxonomic rank (to genus level) together with its ASV ID. The ASVs are color-coded according to the phyla they belong to and plotted according to their effect size, calculated as the levels in samples from experimental microcosms relative to levels in samples from larval rearing pans. Dot sizes correspond to the median centered log-ratio (clr) abundance value for each ASV across rearing pan samples. Dots with a bold red outline represent ASVs that were differentially abundant in both experimental microcosms containing sterile water plus material from cryopreserved stocks and experimental microcosms containing unprocessed water (see S3 Fig).
(TIF)

S5 Fig Rarefaction data from Illumina sequencing of water from a naturally occurring mosquito larval habitat in the field and resulting experimental microcosms. Reads from each water library were sampled starting at 1 sequence per step and increased in increments of 100 until the total number of reads per sample was reached. Lines are colored by sample source (habitat water samples, brown; experimental microcosms containing unprocessed habitat water, tan; experimental microcosms containing sterile water plus material from a given cryopreserved stock, orange). Time of sampling of experimental microcosms is designated by line type (Day 2, long-dash; Day 5, dot-dash).
(TIF)

S6 Fig Differentially abundant ASVs in water samples collected from a naturally occurring mosquito larval habitat in the field and experimental microcosms containing unprocessed habitat water, identified through ALDEx2 testing (ALDEx2, *P* < 0.05; FDR, *P* < 0.05). Each ASV is presented with its lowest annotated taxonomic rank (to genus level) together with its ASV ID. The ASVs are color-coded according to the phyla they belong to and plotted according to their effect size, calculated as the levels in samples from experimental microcosms relative to levels in habitat water samples. Dot sizes correspond to the median centered log-ratio (clr) abundance value for each ASV across habitat water samples. Dots with a bold red outline represent ASVs that were differentially abundant in both experimental microcosms containing unprocessed habitat water and experimental microcosms containing sterile water plus material from cryopreserved stocks (see S7 Fig).
(TIF)

S7 Fig Differentially abundant ASVs in water collected from a naturally occurring mosquito larval habitat in the field and experimental microcosms containing sterile water plus material from cryopreserved stocks, identified through ALDEx2 testing (ALDEx2, *P* < 0.05; FDR, *P* < 0.05). Each ASV is presented with its lowest annotated taxonomic rank (to genus level) together with its ASV ID. The ASVs are color-coded according to the phyla they belong to and plotted according to their effect size, calculated as the levels in samples from experimental microcosms relative to levels in habitat water samples. Dot sizes correspond to the median centered log-ratio (clr) abundance value for each ASV across habitat water samples. Dots with a bold red outline represent ASVs that were differentially abundant in both experimental microcosms containing sterile water plus material from cryopreserved stocks and experimental microcosms containing unprocessed water (see S6 Fig).
(TIF)
