# Supplementary material for: A cryopreservation method to recover laboratory- and field-derived bacterial communities from mosquito larval habitats
Source: PLoS Negl Trop Dis. 2023 Apr 5;17(4):e0011234. doi: 10.1371/journal.pntd.0011234 (PMC10109488; doi:10.1371/journal.pntd.0011234)
Supplement: S1 Table — Asterisks (*) indicate samples that were removed from the dataset prior to downstream analyses. (DOCX) [file pntd.0011234.s001.docx]

| **Table S1.** Sequencing and diversity statistics for 16S rRNA gene amplicon libraries prepared from water collected from conventional larval rearing pans in the laboratory and resulting experimental microcosms. Asterisks (*) indicate samples that were removed from the dataset prior to downstream analyses. | | | | | | |
| --- | --- | --- | --- | --- | --- | --- |
| **Sample ID** | **Sample type** | **Density** | **Time of sampling** | **Total reads** | **Total ASVs** | **Shannon index** |
| 50ml-A | Rearing pan | - | - | 43265 | 61 | 4.1742 |
| 50ml-B | Rearing pan | - | - | 27500 | 65 | 4.2197 |
| 50ml-C | Rearing pan | - | - | 71307 | 58 | 3.8723 |
| 50ml-D | Rearing pan | - | - | 45225 | 70 | 4.2343 |
| 50x-d2-A | Microcosm (unprocessed) | - | Day 2 | 122102 | 376 | 5.3964 |
| 50x-d2-B | Microcosm (unprocessed) | - | Day 2 | 60103 | 94 | 4.5934 |
| 50x-d2-C | Microcosm (unprocessed) | - | Day 2 | 112373 | 440 | 5.5159 |
| 50x-d2-D | Microcosm (unprocessed) | - | Day 2 | 52752 | 85 | 3.9012 |
| 50x-d5-A | Microcosm (unprocessed) | - | Day 5 | 8888 | 47 | 3.1674 |
| 50x-d5-B | Microcosm (unprocessed) | - | Day 5 | 25877 | 93 | 4.3186 |
| 50x-d5-C | Microcosm (unprocessed) | - | Day 5 | 16892 | 63 | 3.4301 |
| 50x-d5-D | Microcosm (unprocessed) | - | Day 5 | 7124 | 64 | 4.4214 |
| 0-d2-A | Microcosm (cryopreserved) | 10^8^ cells per ml | Day 2 | 998 | 17 | 2.8264 |
| 0-d2-B | Microcosm (cryopreserved) | 10^8^ cells per ml | Day 2 | 56898 | 50 | 4.0687 |
| 0-d2-C | Microcosm (cryopreserved) | 10^8^ cells per ml | Day 2 | 50352 | 53 | 3.8461 |
| 0-d2-D | Microcosm (cryopreserved) | 10^8^ cells per ml | Day 2 | 50979 | 57 | 4.2447 |
| 0-d5-A | Microcosm (cryopreserved) | 10^8^ cells per ml | Day 5 | 20128 | 45 | 3.5096 |
| 0-d5-B | Microcosm (cryopreserved) | 10^8^ cells per ml | Day 5 | 36407 | 57 | 3.9757 |
| 0-d5-C | Microcosm (cryopreserved) | 10^8^ cells per ml | Day 5 | 53569 | 48 | 3.6420 |
| 0-d5-D | Microcosm (cryopreserved) | 10^8^ cells per ml | Day 5 | 26264 | 61 | 4.2549 |
| 1-d2-A | Microcosm (cryopreserved) | 10^7^ cells per ml | Day 2 | 44633 | 38 | 3.3959 |
| 1-d2-B | Microcosm (cryopreserved) | 10^7^ cells per ml | Day 2 | 67859 | 46 | 3.5527 |
| 1-d2-C | Microcosm (cryopreserved) | 10^7^ cells per ml | Day 2 | 62482 | 45 | 3.5508 |
| 1-d2-D | Microcosm (cryopreserved) | 10^7^ cells per ml | Day 2 | 56016 | 40 | 3.6281 |
| 1-d5-A | Microcosm (cryopreserved) | 10^7^ cells per ml | Day 5 | 52662 | 47 | 3.6479 |
| 1-d5-B | Microcosm (cryopreserved) | 10^7^ cells per ml | Day 5 | 20921 | 49 | 3.7809 |
| 1-d5-C | Microcosm (cryopreserved) | 10^7^ cells per ml | Day 5 | 33806 | 44 | 3.5116 |
| 1-d5-D | Microcosm (cryopreserved) | 10^7^ cells per ml | Day 5 | 35559 | 52 | 3.7764 |
| 2-d2-A | Microcosm (cryopreserved) | 10^6^ cells per ml | Day 2 | 59358 | 37 | 3.1160 |
| 2-d2-B | Microcosm (cryopreserved) | 10^6^ cells per ml | Day 2 | 43085 | 42 | 3.6196 |
| 2-d2-C | Microcosm (cryopreserved) | 10^6^ cells per ml | Day 2 | 67607 | 41 | 3.3986 |
| 2-d2-D* | Microcosm (cryopreserved) | 10^6^ cells per ml | Day 2 | 28 | - | - |
| 2-d5-A | Microcosm (cryopreserved) | 10^6^ cells per ml | Day 5 | 61484 | 39 | 3.4294 |
| 2-d5-B | Microcosm (cryopreserved) | 10^6^ cells per ml | Day 5 | 37861 | 45 | 3.6205 |
| 2-d5-C | Microcosm (cryopreserved) | 10^6^ cells per ml | Day 5 | 36451 | 37 | 3.2768 |
| 2-d5-D | Microcosm (cryopreserved) | 10^6^ cells per ml | Day 5 | 27671 | 40 | 3.5898 |
| 3-d2-A | Microcosm (cryopreserved) | 10^5^ cells per ml | Day 2 | 31563 | 29 | 3.3386 |
| 3-d2-B | Microcosm (cryopreserved) | 10^5^ cells per ml | Day 2 | 53234 | 37 | 3.1552 |
| 3-d2-C | Microcosm (cryopreserved) | 10^5^ cells per ml | Day 2 | 38611 | 33 | 3.5208 |
| 3-d2-D | Microcosm (cryopreserved) | 10^5^ cells per ml | Day 2 | 61732 | 39 | 3.4932 |
| 3-d5-A | Microcosm (cryopreserved) | 10^5^ cells per ml | Day 5 | 57258 | 35 | 3.4729 |
| 3-d5-B | Microcosm (cryopreserved) | 10^5^ cells per ml | Day 5 | 41448 | 43 | 3.6010 |
| 3-d5-C | Microcosm (cryopreserved) | 10^5^ cells per ml | Day 5 | 34395 | 35 | 3.2676 |
| 3-d5-D | Microcosm (cryopreserved) | 10^5^ cells per ml | Day 5 | 32762 | 40 | 3.5156 |
| 4-d2-A | Microcosm (cryopreserved) | 10^4^ cells per ml | Day 2 | 51176 | 31 | 3.2377 |
| 4-d2-B | Microcosm (cryopreserved) | 10^4^ cells per ml | Day 2 | 49723 | 31 | 3.2008 |
| 4-d2-C* | Microcosm (cryopreserved) | 10^4^ cells per ml | Day 2 | 41 | - | - |
| 4-d2-D | Microcosm (cryopreserved) | 10^4^ cells per ml | Day 2 | 62944 | 37 | 3.4669 |
| 4-d5-A | Microcosm (cryopreserved) | 10^4^ cells per ml | Day 5 | 38359 | 32 | 3.4570 |
| 4-d5-B | Microcosm (cryopreserved) | 10^4^ cells per ml | Day 5 | 34889 | 31 | 3.3242 |
| 4-d5-C | Microcosm (cryopreserved) | 10^4^ cells per ml | Day 5 | 63315 | 34 | 3.3751 |
| 4-d5-D | Microcosm (cryopreserved) | 10^4^ cells per ml | Day 5 | 25329 | 37 | 3.4865 |
| 5-d2-A | Microcosm (cryopreserved) | 10^3^ cells per ml | Day 2 | 54637 | 27 | 3.1312 |
| 5-d2-B | Microcosm (cryopreserved) | 10^3^ cells per ml | Day 2 | 57438 | 30 | 3.0905 |
| 5-d2-C | Microcosm (cryopreserved) | 10^3^ cells per ml | Day 2 | 60946 | 29 | 3.2835 |
| 5-d2-D | Microcosm (cryopreserved) | 10^3^ cells per ml | Day 2 | 44747 | 27 | 3.3447 |
| 5-d5-A | Microcosm (cryopreserved) | 10^3^ cells per ml | Day 5 | 35667 | 30 | 3.2233 |
| 5-d5-B | Microcosm (cryopreserved) | 10^3^ cells per ml | Day 5 | 43450 | 37 | 3.1519 |
| 5-d5-C | Microcosm (cryopreserved) | 10^3^ cells per ml | Day 5 | 52643 | 32 | 3.2932 |
| 5-d5-D | Microcosm (cryopreserved) | 10^3^ cells per ml | Day 5 | 45081 | 32 | 3.4238 |
