# Supplementary material for: A cryopreservation method to recover laboratory- and field-derived bacterial communities from mosquito larval habitats
Source: PLoS Negl Trop Dis. 2023 Apr 5;17(4):e0011234. doi: 10.1371/journal.pntd.0011234 (PMC10109488; doi:10.1371/journal.pntd.0011234)
Supplement: S2 Table — (DOCX) [file pntd.0011234.s002.docx]

| **Table S2.** Sequencing and diversity statistics for 16S rRNA gene amplicon libraries prepared from water collected from a naturally occurring mosquito larval habitat in the field and resulting experimental microcosms. | | | | | | |
| --- | --- | --- | --- | --- | --- | --- |
| **Sample ID** | **Sample type** | **Density** | **Time of sampling** | **Total reads** | **Total ASVs** | **Shannon index** |
| F-50ml-A | Habitat water | - | - | 3964 | 179 | 6.1297 |
| F-50ml-B | Habitat water | - | - | 38836 | 798 | 7.2439 |
| F-50ml-C | Habitat water | - | - | 9463 | 295 | 6.5045 |
| F-50ml-D | Habitat water | - | - | 10684 | 367 | 6.6975 |
| F-50x-d2-A | Microcosm (unprocessed) | - | Day 2 | 145719 | 312 | 5.6223 |
| F-50x-d2-B | Microcosm (unprocessed) | - | Day 2 | 90965 | 275 | 5.8426 |
| F-50x-d2-C | Microcosm (unprocessed) | - | Day 2 | 89703 | 288 | 5.8295 |
| F-50x-d2-D | Microcosm (unprocessed) | - | Day 2 | 59059 | 239 | 5.8823 |
| F-50x-d5-A | Microcosm (unprocessed) | - | Day 5 | 45717 | 315 | 6.0728 |
| F-50x-d5-B | Microcosm (unprocessed) | - | Day 5 | 17268 | 244 | 6.7062 |
| F-50x-d5-C | Microcosm (unprocessed) | - | Day 5 | 74564 | 387 | 5.9063 |
| F-50x-d5-D | Microcosm (unprocessed) | - | Day 5 | 34632 | 247 | 5.4338 |
| F-0-d2-A | Microcosm (cryopreserved) | 10^6^ cells per ml | Day 2 | 62480 | 75 | 3.5792 |
| F-0-d2-B | Microcosm (cryopreserved) | 10^6^ cells per ml | Day 2 | 54280 | 67 | 3.4459 |
| F-0-d2-C | Microcosm (cryopreserved) | 10^6^ cells per ml | Day 2 | 24488 | 57 | 3.2725 |
| F-0-d2-D | Microcosm (cryopreserved) | 10^6^ cells per ml | Day 2 | 30310 | 72 | 3.9956 |
| F-0-d5-A | Microcosm (cryopreserved) | 10^6^ cells per ml | Day 5 | 48528 | 92 | 3.6742 |
| F-0-d5-B | Microcosm (cryopreserved) | 10^6^ cells per ml | Day 5 | 30520 | 57 | 3.3095 |
| F-0-d5-C | Microcosm (cryopreserved) | 10^6^ cells per ml | Day 5 | 152484 | 111 | 3.5537 |
| F-0-d5-D | Microcosm (cryopreserved) | 10^6^ cells per ml | Day 5 | 48067 | 84 | 4.0770 |
| F-1-d2-A | Microcosm (cryopreserved) | 10^5^ cells per ml | Day 2 | 20678 | 21 | 3.0125 |
| F-1-d2-B | Microcosm (cryopreserved) | 10^5^ cells per ml | Day 2 | 46650 | 25 | 3.2540 |
| F-1-d2-C | Microcosm (cryopreserved) | 10^5^ cells per ml | Day 2 | 18065 | 25 | 2.9398 |
| F-1-d2-D | Microcosm (cryopreserved) | 10^5^ cells per ml | Day 2 | 34546 | 26 | 2.5901 |
| F-1-d5-A | Microcosm (cryopreserved) | 10^5^ cells per ml | Day 5 | 49969 | 38 | 2.9305 |
| F-1-d5-B | Microcosm (cryopreserved) | 10^5^ cells per ml | Day 5 | 57471 | 36 | 3.0436 |
| F-1-d5-C | Microcosm (cryopreserved) | 10^5^ cells per ml | Day 5 | 67926 | 36 | 2.7067 |
| F-1-d5-D | Microcosm (cryopreserved) | 10^5^ cells per ml | Day 5 | 57401 | 33 | 2.4124 |
| F-2-d2-A | Microcosm (cryopreserved) | 10^4^ cells per ml | Day 2 | 29184 | 11 | 1.7388 |
| F-2-d2-B | Microcosm (cryopreserved) | 10^4^ cells per ml | Day 2 | 61850 | 22 | 2.8832 |
| F-2-d2-C | Microcosm (cryopreserved) | 10^4^ cells per ml | Day 2 | 97008 | 14 | 1.4067 |
| F-2-d2-D | Microcosm (cryopreserved) | 10^4^ cells per ml | Day 2 | 36429 | 13 | 2.4710 |
| F-2-d5-A | Microcosm (cryopreserved) | 10^4^ cells per ml | Day 5 | 44047 | 16 | 2.2003 |
| F-2-d5-B | Microcosm (cryopreserved) | 10^4^ cells per ml | Day 5 | 41637 | 27 | 2.4174 |
| F-2-d5-C | Microcosm (cryopreserved) | 10^4^ cells per ml | Day 5 | 35402 | 13 | 1.8279 |
| F-2-d5-D | Microcosm (cryopreserved) | 10^4^ cells per ml | Day 5 | 35949 | 17 | 2.7682 |
| F-3-d2-A | Microcosm (cryopreserved) | 10^3^ cells per ml | Day 2 | 43035 | 5 | 0.0089 |
| F-3-d2-B | Microcosm (cryopreserved) | 10^3^ cells per ml | Day 2 | 36831 | 13 | 0.7838 |
| F-3-d2-C | Microcosm (cryopreserved) | 10^3^ cells per ml | Day 2 | 47713 | 19 | 1.8455 |
| F-3-d2-D | Microcosm (cryopreserved) | 10^3^ cells per ml | Day 2 | 15000 | 5 | 0.0602 |
| F-3-d5-A | Microcosm (cryopreserved) | 10^3^ cells per ml | Day 5 | 56109 | 5 | 0.0455 |
| F-3-d5-B | Microcosm (cryopreserved) | 10^3^ cells per ml | Day 5 | 24035 | 9 | 1.9925 |
| F-3-d5-C | Microcosm (cryopreserved) | 10^3^ cells per ml | Day 5 | 34929 | 19 | 1.4651 |
| F-3-d5-D | Microcosm (cryopreserved) | 10^3^ cells per ml | Day 5 | 47450 | 5 | 1.4273 |
