# Supplementary figures and images for: A cryopreservation method to recover laboratory- and field-derived bacterial communities from mosquito larval habitats
Source: PLoS Negl Trop Dis. 2023 Apr 5;17(4):e0011234. doi: 10.1371/journal.pntd.0011234 (PMC10109488; doi:10.1371/journal.pntd.0011234)

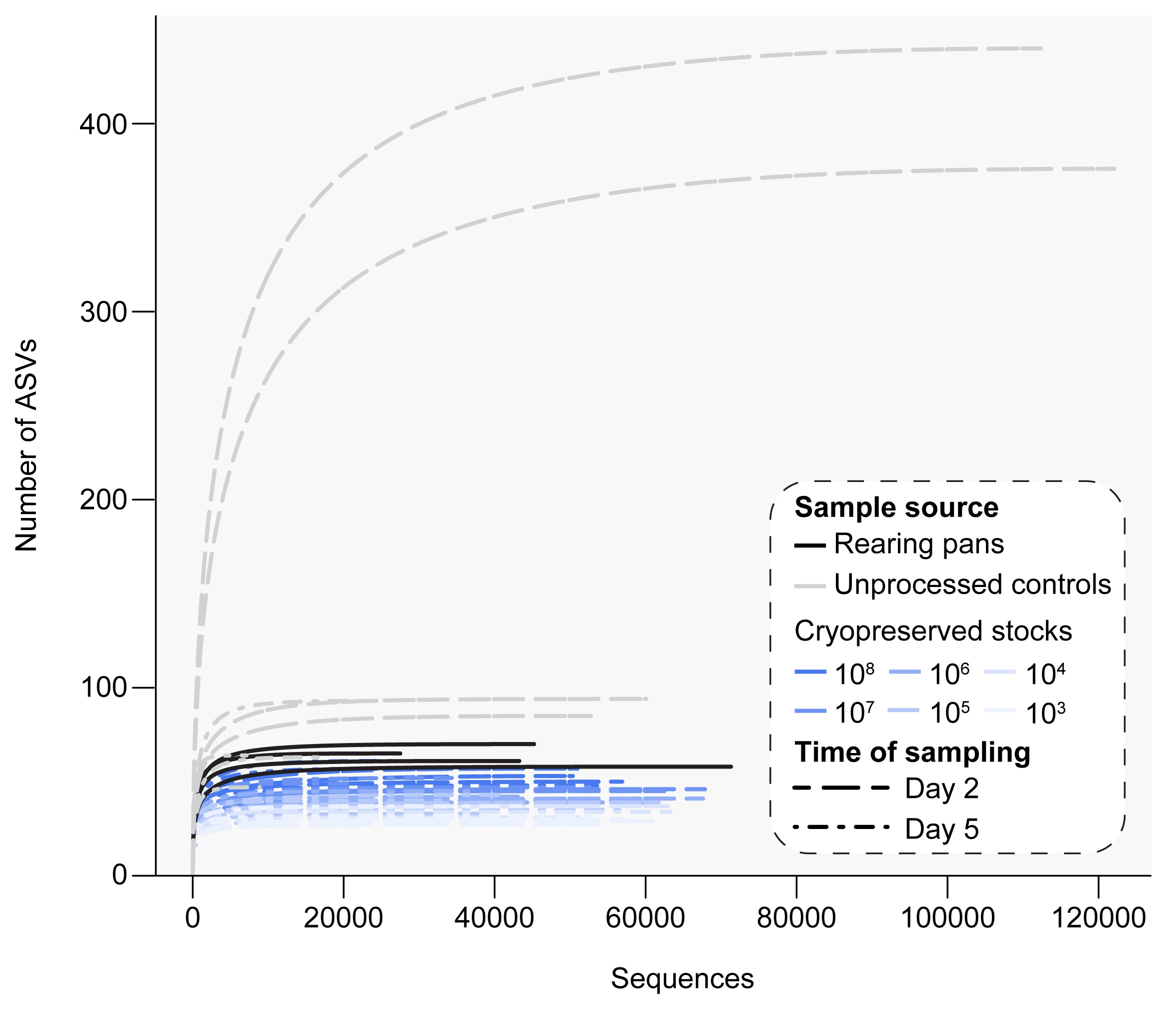

Supplement: S1 Fig — Reads from each water library were sampled starting at 1 sequence per step and increased in increments of 100 until the total number of reads per sample was reached. Lines are colored by sample source (rearing pans, black; experimental microcosms containing unprocessed water, grey; experimental microcosms containing sterile water plus material from a given cryopreserved stock, blue). Time of sampling of experimental microcosms is designated by line type (Day 2, long-dash; Day 5, dot-dash). (TIF) [file pntd.0011234.s003.tif]

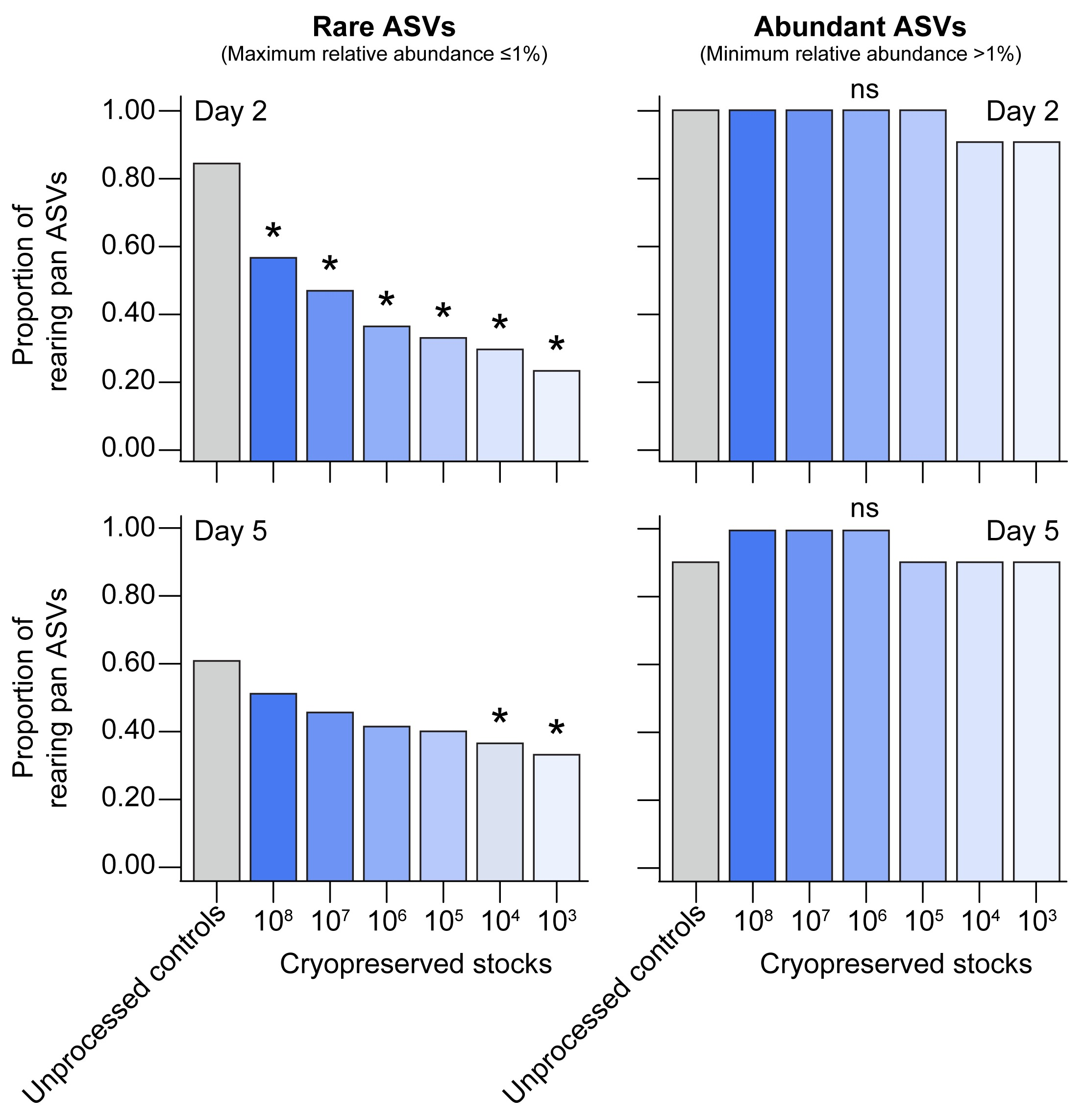

Supplement: S2 Fig — Proportion of rare (left) and abundant (right) ASVs found in at least one larval rearing pan that were detected in experimental microcosms containing unprocessed water or sterile water plus material from a given cryopreserved stock. An ASV was considered “rare” if it had a maximum relative abundance ≤1% across the four larval rearing pans we sampled, while ASVs with a minimum relative abundance >1% were considered “abundant”. Asterisks (*) indicate significant differences between experimental microcosms generated using cryopreserved stocks relative to unprocessed controls as determined by paired Fisher’s exact tests with Bonferroni correction (P < 0.05). (TIF) [file pntd.0011234.s004.tif]

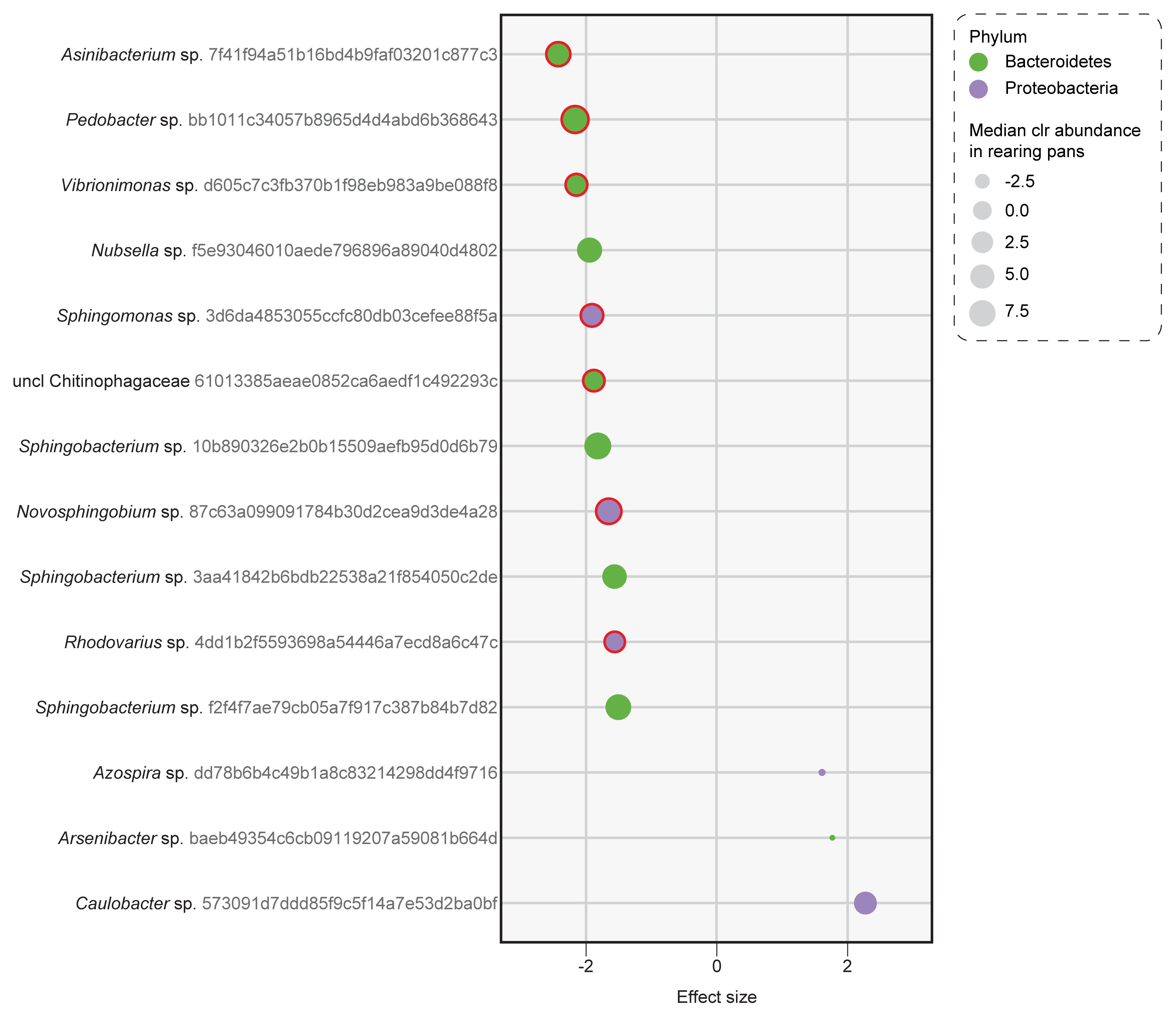

Supplement: S3 Fig — Each ASV is presented with its lowest annotated taxonomic rank (to genus level) together with its ASV ID. The ASVs are color-coded according to the phyla they belong to and plotted according to their effect size, calculated as the levels in samples from experimental microcosms relative to levels in samples from larval rearing pans. Dot sizes correspond to the median centered log-ratio (clr) abundance value for each ASV across rearing pan samples. Dots with a bold red outline represent ASVs that were differentially abundant in both experimental microcosms containing unprocessed water and experimental microcosms containing sterile water plus material from cryopreserved stocks (see S4 Fig). (TIF) [file pntd.0011234.s005.tif]

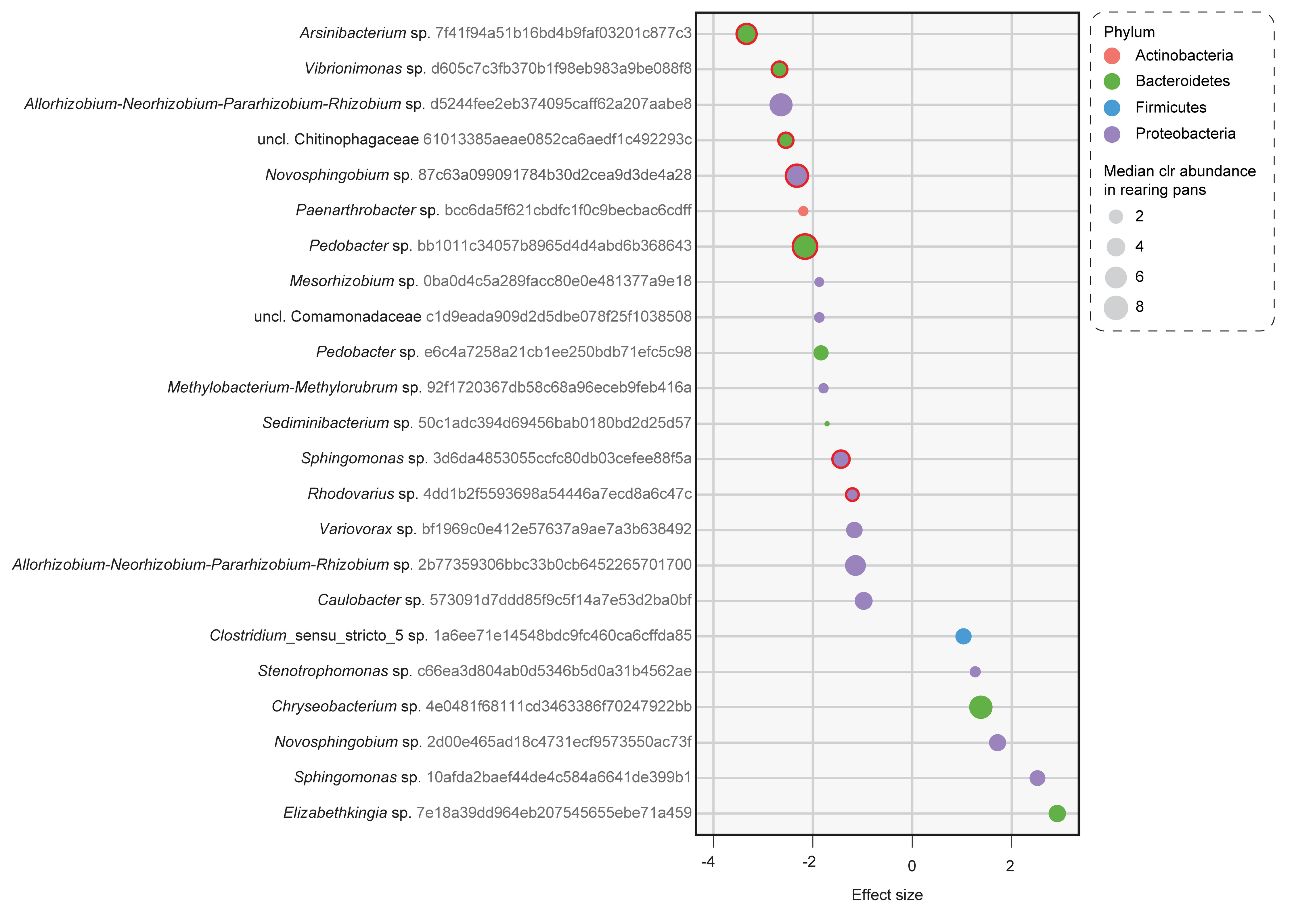

Supplement: S4 Fig — Each ASV is presented with its lowest annotated taxonomic rank (to genus level) together with its ASV ID. The ASVs are color-coded according to the phyla they belong to and plotted according to their effect size, calculated as the levels in samples from experimental microcosms relative to levels in samples from larval rearing pans. Dot sizes correspond to the median centered log-ratio (clr) abundance value for each ASV across rearing pan samples. Dots with a bold red outline represent ASVs that were differentially abundant in both experimental microcosms containing sterile water plus material from cryopreserved stocks and experimental microcosms containing unprocessed water (see S3 Fig). (TIF) [file pntd.0011234.s006.tif]

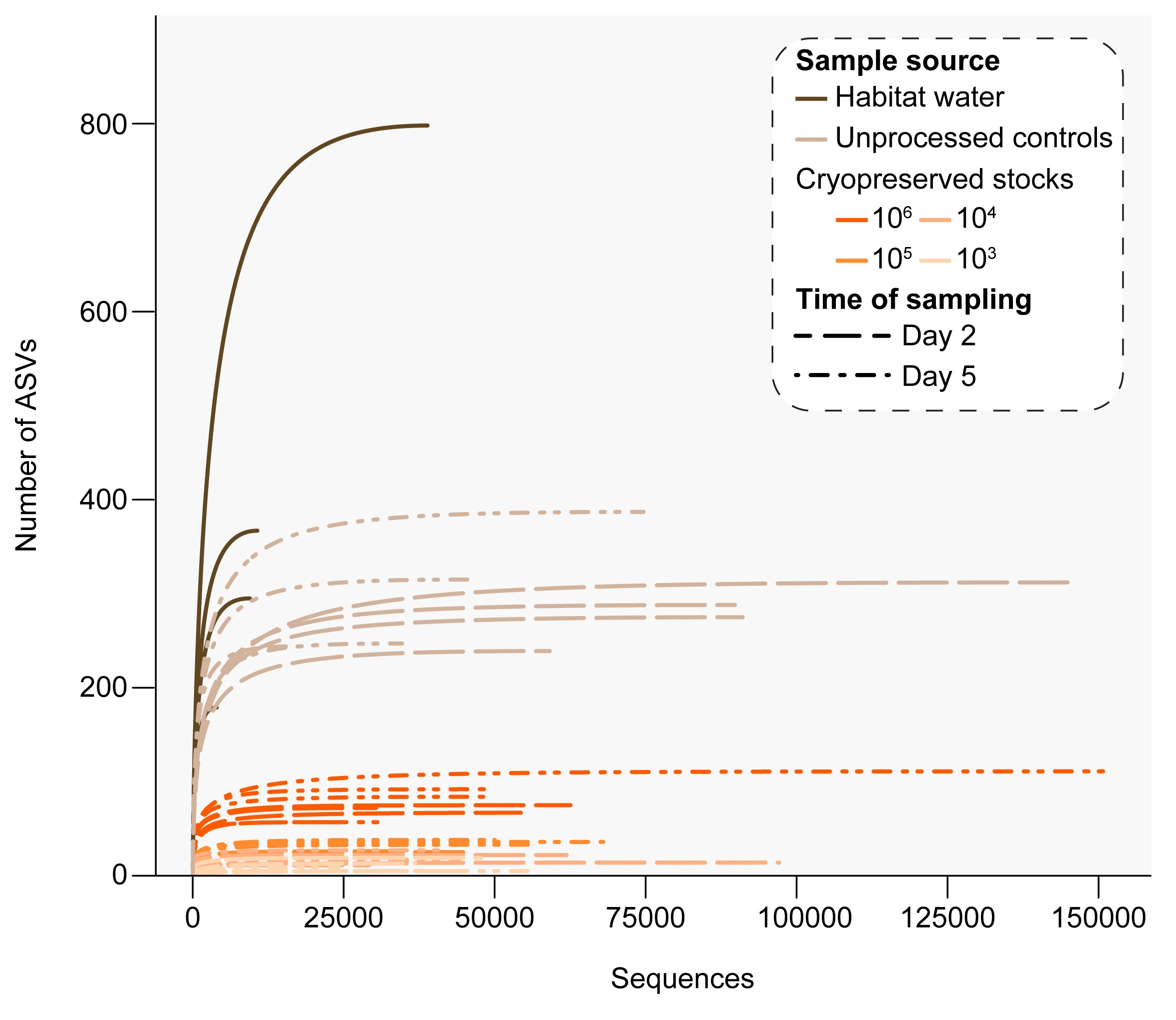

Supplement: S5 Fig — Reads from each water library were sampled starting at 1 sequence per step and increased in increments of 100 until the total number of reads per sample was reached. Lines are colored by sample source (habitat water samples, brown; experimental microcosms containing unprocessed habitat water, tan; experimental microcosms containing sterile water plus material from a given cryopreserved stock, orange). Time of sampling of experimental microcosms is designated by line type (Day 2, long-dash; Day 5, dot-dash). (TIF) [file pntd.0011234.s007.tif]

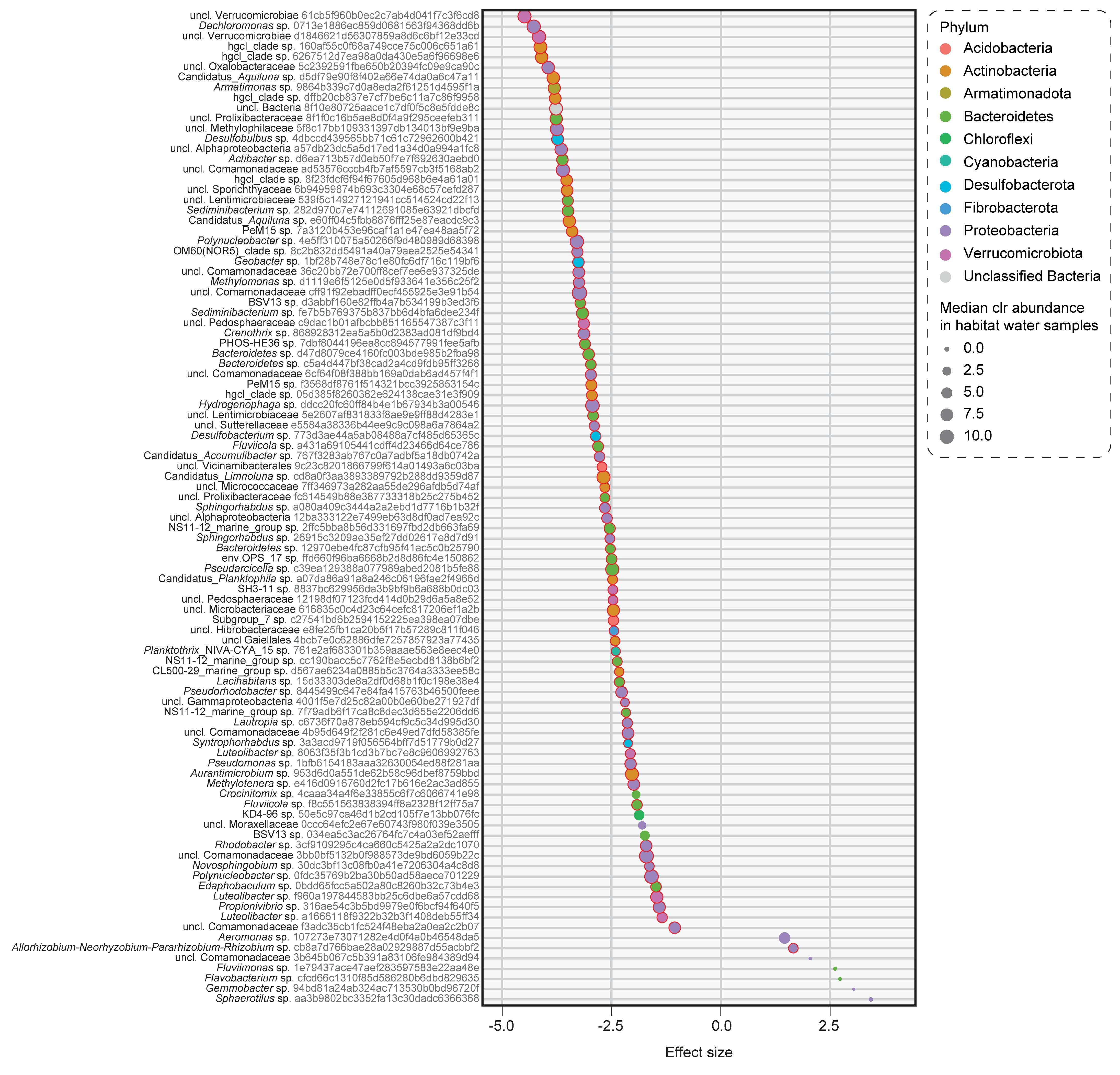

Supplement: S6 Fig — Each ASV is presented with its lowest annotated taxonomic rank (to genus level) together with its ASV ID. The ASVs are color-coded according to the phyla they belong to and plotted according to their effect size, calculated as the levels in samples from experimental microcosms relative to levels in habitat water samples. Dot sizes correspond to the median centered log-ratio (clr) abundance value for each ASV across habitat water samples. Dots with a bold red outline represent ASVs that were differentially abundant in both experimental microcosms containing unprocessed habitat water and experimental microcosms containing sterile water plus material from cryopreserved stocks (see S7 Fig). (TIF) [file pntd.0011234.s008.tif]

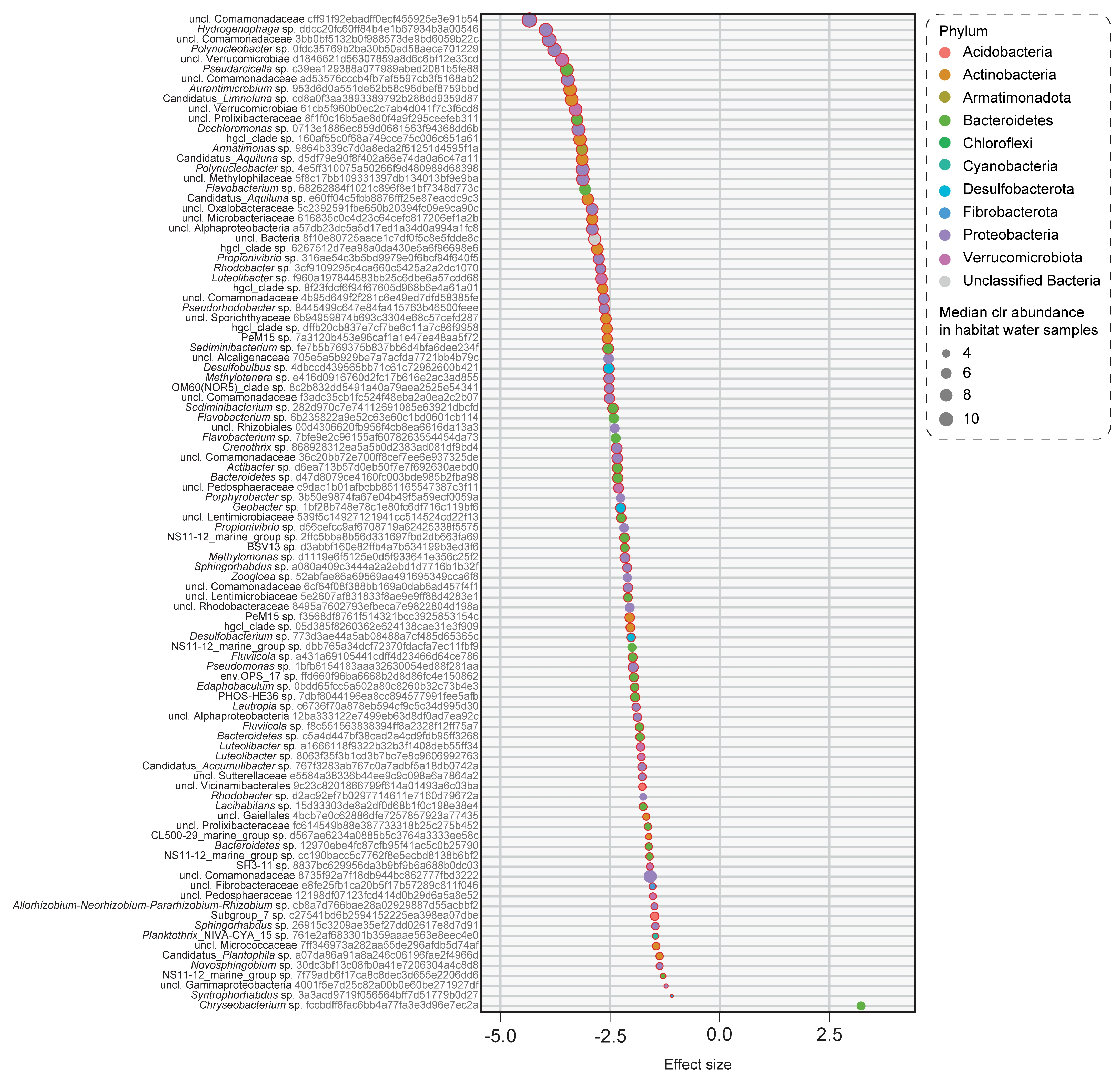

Supplement: S7 Fig — Each ASV is presented with its lowest annotated taxonomic rank (to genus level) together with its ASV ID. The ASVs are color-coded according to the phyla they belong to and plotted according to their effect size, calculated as the levels in samples from experimental microcosms relative to levels in habitat water samples. Dot sizes correspond to the median centered log-ratio (clr) abundance value for each ASV across habitat water samples. Dots with a bold red outline represent ASVs that were differentially abundant in both experimental microcosms containing sterile water plus material from cryopreserved stocks and experimental microcosms containing unprocessed water (see S6 Fig). (TIF) [file pntd.0011234.s009.tif]
